# Opposite trends in the genus *Monsonia* (Geraniaceae): specialization in the African deserts and range expansions throughout eastern Africa

**DOI:** 10.1038/s41598-017-09834-6

**Published:** 2017-08-29

**Authors:** Sara García-Aloy, Isabel Sanmartín, Gudrun Kadereit, Daniel Vitales, Ana María Millanes, Cristina Roquet, Pablo Vargas, Marisa Alarcón, Juan José Aldasoro

**Affiliations:** 1 Institut Botànic de Barcelona (IBB-CSIC-ICUB), Passeig del Migdia s/n, Parc de Montjuïc, E-08038 Barcelona, Spain; 2 Real Jardín Botánico, (RJB-CSIC), Plaza de Murillo 2, E-28014 Madrid, Spain; 3 Institut für Molekulare Physiologie, Johannes Gutenberg-Universität Mainz, D-55099 Mainz, Germany; 4 Universidad Rey Juan Carlos (URJC), C/ Tulipán s.n., E-, 28933 Móstoles, Spain; 5 Laboratoire d’Écologie Alpine, CNRS UMR 5553, Université Grenoble Alpes, BP 53, F-38041 Grenoble Cedex 9, France

## Abstract

The African Austro-temperate Flora stands out by its important species richness. A distinctive element of this flora is *Monsonia* (Geraniaceae), mostly found in the Namib-Karoo but also in the Natal-Drakensberg, the Somalian Zambezian and the Saharo-Arabian regions. Here, we reconstruct the evolution and biogeographic history of *Monsonia* based on nuclear and plastid markers, and examine the role of morphological and niche evolution in its diversification using species distribution modeling and macroevolutionary models. Our results indicate that *Monsonia* first diversified in the Early Miocene c.21 Ma, coinciding with the start of desertification in southwestern Africa. An important diversification occurred c. 4–6 Ma, after a general cooling trend in western South Africa and the rising of the Eastern African Mountains. The resulting two main lineages of *Monsonia* are constituted by: (1) Namib-Karoo succulents, and (2) herbs of the Natal-Drakensberg plus three species that further colonised steppes in north and eastern Africa. The highest diversity of *Monsonia* is found in the Namib-Karoo coastal belt, within a mosaic-like habitat structure. Diversification was likely driven by biome shifts and key innovations such as water-storing succulent stems and anemochorous fruits. In contrast, and unlike other arid-adapted taxa, all species of *Monsonia* share a C_3_ metabolism.

## Introduction

The African Austro-temperate Flora (AAF) is characterized by high species richness and levels of endemism, and constitutes one of the world’s richest areas in plant diversity^1, 2^. The AAF includes the Natal grasslands and savannas, the Fynbos, the Namib Desert, the Nama Karoo, and the Succulent Karoo biomes^3^, the latter harbouring an extraordinarily diverse arid flora. Several studies have linked the remarkable plant diversity levels found in the AAF to the presence of recent radiations^4^ and persistence in older lineages^5^, suggesting a complex evolutionary pattern^6, 7^. Patterns of diversity in the AAF have been alternatively linked to abiotic or biotic factors, including climate gradients, topographical complexity, niche subdivision, and the appearance of morphological novelties or key innovations^8^.

The Neogene history of Africa is one of aridification and desertification. Waves of aridification, interspersed with periods of more humid climate, have profoundly impacted the vegetation composition of this continent, from a former subtropical “Gondwanan” flora to one dominated by arid and semiarid elements^9^. The Cenozoic northeastward drift of the continent towards the equatorial zone, the uplift of eastern Africa starting in the Eocene, and the closing of the Tethys Sea after the collision with Eurasia, are some of the main causes behind this aridification trend^10, 11^. A major aridification episode began in the early Miocene (c. 17 Mya), in the Namib and the Kalahari deserts^11^, which was followed by other drought events in the Namib-Karoo, the Sahara and the Eastern Arc Mountains in the later Miocene (8–9 Mya)^9, 12^. Aridification in the Namib Desert climaxed with the upwelling of deep waters –the Benguela Current c. 8–10 Mya^13^. The Namib Desert stretches from southern Angola through Namibia into South Africa (i.e. the Succulent Karoo) with a variable width (80–200 km) and gradual rise from the coast to the Namib Escarpment. These climate changes generated a wealth of new habitats across southwestern Africa, and triggered speciation in drought-tolerant geophytes, succulents, and species with CAM/C_4_ metabolism^1, 14, 15^.

Orogenic events also impacted biodiversity; for example, the main uplift of the Drakensberg Mountains at the Miocene-Pliocene boundary (5.5 Ma) coincided with an increase in diversification rates in certain groups^16^. The uplift of the East African Rift System during the Plio-Pleistocene also underlies diversification rate shifts in other groups^11, 15^. Between these mountain ranges and the West-Central Guineo-Congolian region is the “African Arid Corridor” (AAC) or the “Arid Track”, a corridor that connects South Africa with other regions, such as the Great Rift, the North African savannas and the Sahara Desert^17^.

**Figure 1:**
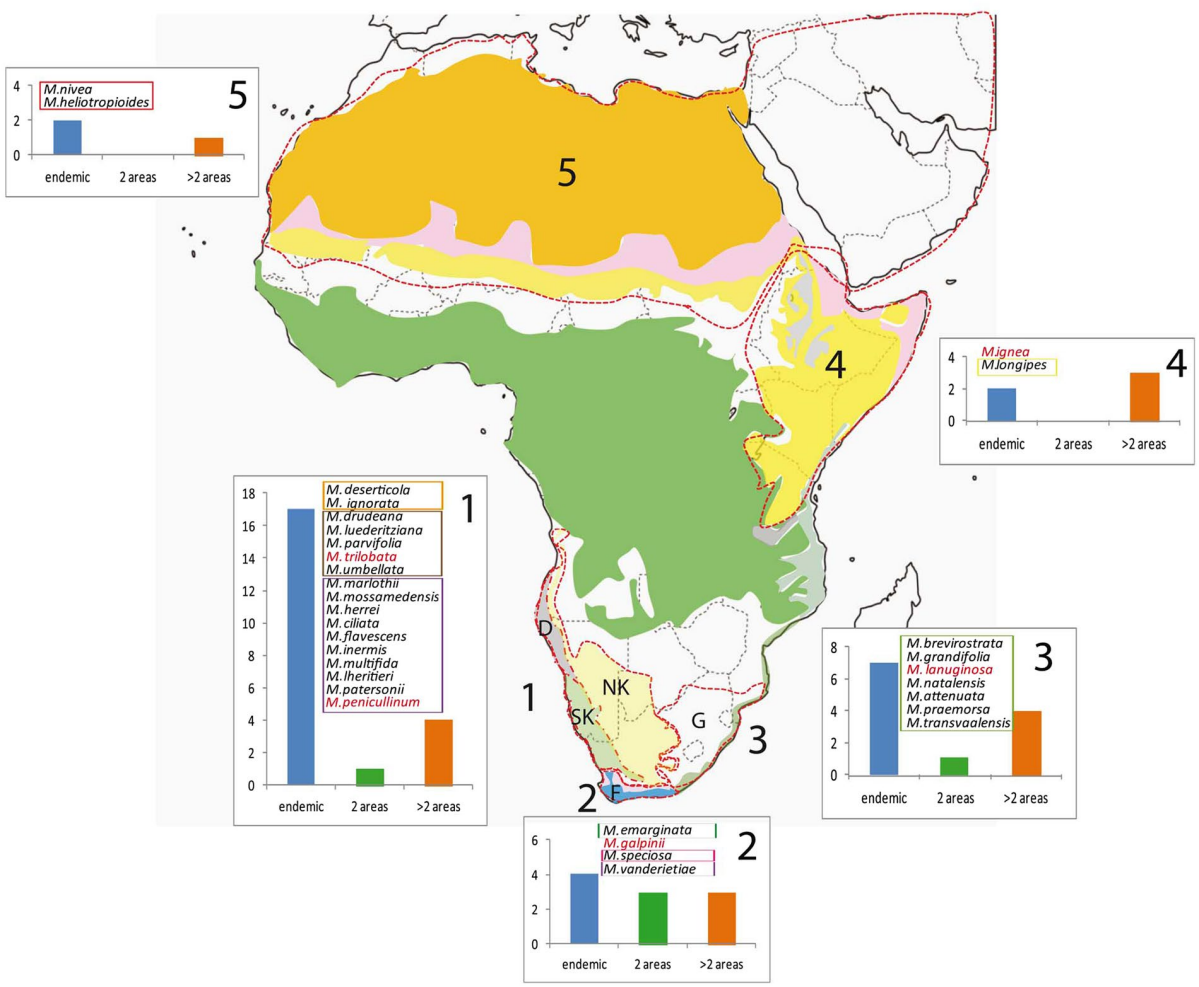
Distribution of species of *Monsonia* in five endemism areas in Africa. Colours indicate the main vegetation zones (map created using software Adobe Illustrator CS4, https://www.adobe.com). The information was obtained from Wikipedia, Plana, 2004 and Senut *et al*.^11^. Names in red indicate species not sampled in this study. The graphics show the number of endemics (blue bar), species shared with another area (green bar), and widespread species (i.e., occurring in more than two areas, orange bar). The coloured square with a letter indicates the phylogenetic adscription of each species (see Fig. 2). The letters in each area indicate the main biomes: D: Namib Desert, SK: Succulent Karoo, NK: Nama Karoo, F: Fynbos, G: Natal Grassslands.

The genus *Monsonia* is a medium sized genus (39 species) mostly distributed in the AAF, with considerable variation in ecological requirements, life forms (annuals, perennials and succulents) and dispersal strategies (anemochory and barochory). *Monsonia* species are adapted to two major habitat types: (1) arid and hyper-arid habitats, and (2) grasslands, fynbos, savannas, and disturbed habitats. Globally, *Monsonia* is distributed in five main areas: (i) the area formed by the Namib Desert, the Succulent Karoo and the Nama-Karoo, (17 endemics), (ii) the Cape (4 endemics), (iii) the Natal-Drakensberg area (7 endemics), (iv) the seasonally arid Somalian and Zambezian Floras (2 endemics), and (v) the Saharo-Arabian arid Flora (2 endemics) (Figs 1 and S1). Up to 84.6% of *Monsonia* species are endemic to one of these areas (Fig. 1). There are many contrasting biogeographic patterns in southwestern Africa: some species exhibit geographically restricted distributions in the coastal belts of the Succulent Karoo and the Namib Desert, other species occupy larger areas in the Succulent Karoo plus the Nama Karoo, while a few ones also extend their ranges into grasslands and savannas of north and east Africa (Fig. 1). Though edaphic complexity has also played a role in the diversification of many South African desert groups, we did not include in this study soil data due to a lack of accurate information for all species of *Monsonia*.

As mentioned above, species of *Monsonia* are characterized by a variety of root types and stem arrangements to maximize productivity in water-limited habitats (i.e., by buffering water availability^18–20^. In other succulent African plants (e.g., *Pelargonium*), measurement of carbon isotope ratios has indicated a flexible C_3_ photosynthesis, with plasticity between CAM and C_3_ metabolism^21^. So far, only two species of *Monsonia* have been studied for CO_2_ fixation patterns (using δ^13^C method), *M*. *mossamedensis* and *M*. *crassicaule*, suggesting a C_3_ metabolism^22^. The desert-adapted species are either rhizomatous perennials that accumulate water in their rhizomes during dry periods, or succulent perennials that store it in their stems. Many desert species present a reduced foliar surface and stomata, which may close during the day. Grassland species are perennial rhizomatous herbs, and less frequently, annuals. Fruit dispersal is either barochorous trypanocarpic, which is a dispersal mechanism that prevents seed dispersal and predation with hygroscopic fruit burying near to the parent plants, or anemochorous (wind dispersal), which is more advantageous way in arid habitats, but at the cost of the loss of a percentage of seeds^23^. Consequently, changes in life-form (succulence, dispersal form) and metabolism type (C_3_, CAM) likely drove the diversification of *Monsonia*, enabling the colonization of novel biomes, such as arid environments that became dominant in the Late Miocene.

Adaptive radiations have often been linked to “key innovations” – the appearance of morphological novelties enabling a lineage to colonize a new environment – and “key opportunities”, events of niche evolution that drive rapid speciation through ecological release^5^. In this study, we investigate whether the appearance of key innovations related to succulence, dispersal and metabolism type (C_3_, CAM) drove the diversification of *Monsonia*, enabling the colonization of novel biomes, such as arid environments that became dominant in the Late Miocene. Restricted gene flow among populations has also been proposed as explanation for the high plant diversity levels in the Namib-Karoo region^24^, which might also apply to *Monsonia*, given the restricted distributions of some species. With its variety of life forms and adaptations to water drought, and its presence in both arid habitats and other semi-arid ecotypes, such as grasslands, fynbos, etc., *Monsonia*, represents an ideal group to examine the relative contribution of these two evolutionary mechanisms (key innovations and ecological evolution) in the diversification of the AAF flora. Additionally, it also offers the opportunity to examine the role played by niche evolution versus niche conservatism (adaptation to arid environments) in the evolution of the African arid flora and on the origins of the Arid Track.

Concretely, our specific goals are: (1) to build a dated phylogenetic tree of *Monsonia* with a nearly complete taxon sampling; (2) to infer the biogeographic origins of *Monsonia* and subsequent dispersal patterns; (3) to assess evidence for a radiation of the genus, identify where and when this took place, (4) to evaluate the role of key innovations (lifeform, CAM/C_3_ metabolism and dispersal type) and climatic niche evolution in the diversification of the genus.

## Results

### Phylogeny, diversification analyses and biogeographic reconstructions

**Figure 2:**
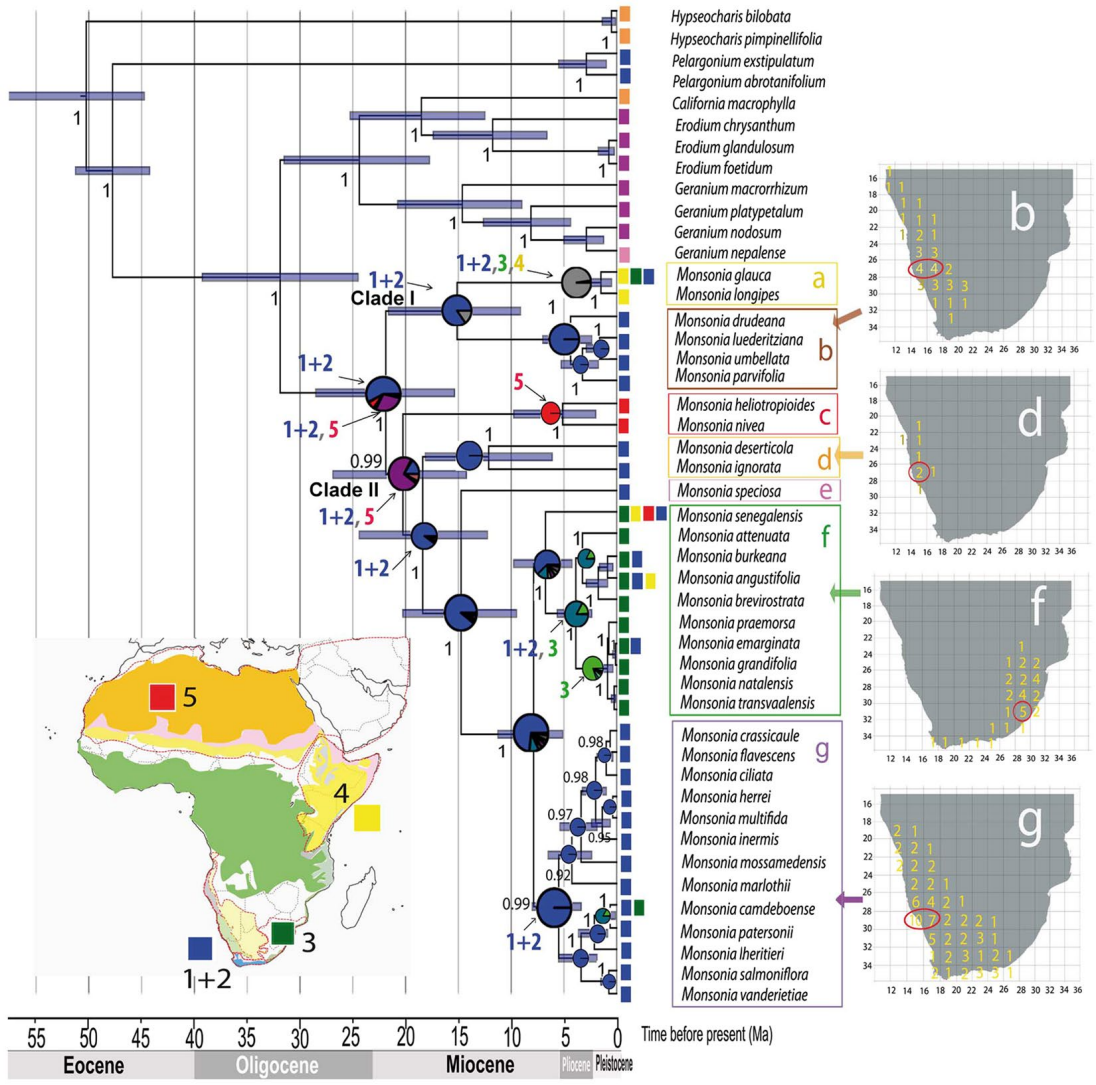
Maximum clade credibility (MCC) tree from the BEAST analysis of plastid and nuclear markers with ancestrsal areas reconstructed using (DEC). Areas are: blue) Namib-Karoo plus the Cape (1 + 2); green) Natal-Drakensberg (3); yellow) Eastern Africa (4); red) Sahara, Arabia and W Asia (5); orange) America; purple) Eurasia. Maps on the right indicate the number of endemic species ascribed to each clade by grid cell of 2° of latitude × 2° longitude. The map with the vegetation zones is a simplification of the map in Fig. 1 generated with the software Adobe Illustrator CS4 (https://www.adobe.com). The maps on the right of the figure were also generated with the software Adobe Illustrator CS4 (https://www.adobe.com).

**Figure 3:**
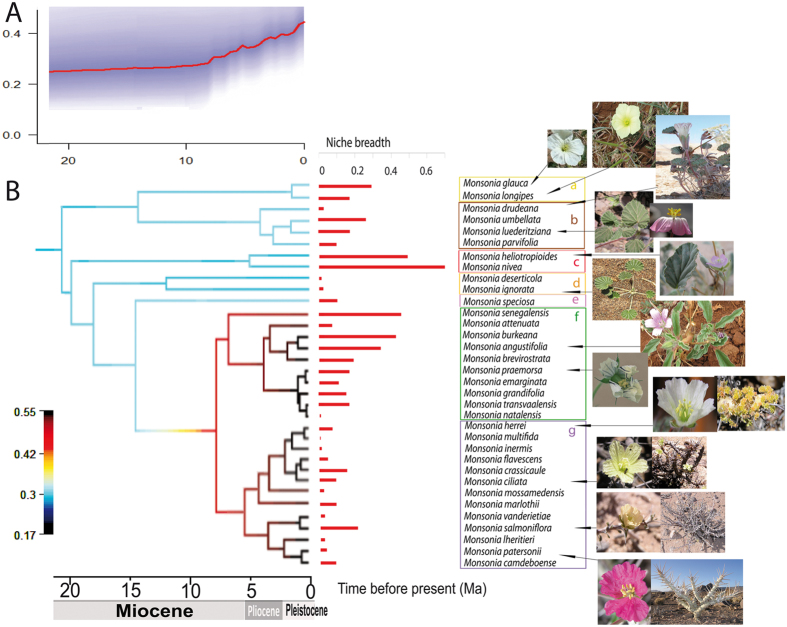
BAMM phylogenetic reconstruction using nuclear and plastid sequences. (**A**) Diversification rate through time (inside the square) and BAMM phylorate plot showing changes in net diversification rates across branches on the MCC tree. (**B**) Niche breadth for each species obtained with ENMtools. All photographs were taken by the co-authors.

Divergence times estimates inferred with BEAST v. 1.7.5 on the concatenated partitioned plastid-nuclear dataset^25^ (Fig. 2) showed two primary clades: Clade I, with only two small subclades: (a) and (b), and Clade II which includes several nested subclades: (c), (d), (e), and the core formed by 23 species divided into two groups, one formed by herbs (f) and the other by succulents (g). All subclades were recovered by both the nuclear and plastid datasets, except for the position of subclade (d) (*M*. *deserticola* and *M*. *ignorata*), which in the nuclear dataset appears as sister to subclades (a) and (b) (Figs S2 and S3). Divergence of *Monsonia* from the genus *Pelargonium* was dated in the Late Eocene-Early Oligocene (c. 30 Ma). *Monsonia* crown-age (the split between Clade I and II) was inferred in the Early Miocene (22 Ma; CI 15.2–28.6), with divergence of main subclades dated in the Early-Mid Miocene period (15–20 Ma). The main exception is the split between the species-rich subclades (f) and (g), which is dated in the Late Miocene (c. 5–12 Ma). DEC biogeographic analysis on the BEAST maximum clade credibility (MCC) tree (Fig. 2) reconstructed the Namib-Karoo-Cape (areas 1 + 2) as the most likely origin for the ancestor of *Monsonia*, with a widespread range comprising the Namib-Karoo-Cape (areas 1 + 2) and the Saharo-Arabian deserts (5) inferred as the second most likely. The ancestor of Clade I (subclades a-b) originated in the same region: the deserts of South Africa followed by a dispersal event to the Great Rift. Clade II (subclades c-g) presents a more complex colonization history: the ancestor was probably widely distributed in the Namib-Karoo-Cape and Saharo-Arabian deserts, while all descendant lineages showed a narrower distribution either in the Saharo-Arabian deserts (subclade c) or in the Namib-Karoo-Cape (subclades d-g). Within subclade (f) an event of range expansion into the Natal-Drakensberg was reconstructed. Diversification rate analyses in BAMM suggested a significant acceleration in diversification rates at the most-recent common ancestor of clades (f) and (g) at the Pliocene-Miocene boundary (Fig. 3).

### Species distribution models, niche breadth and overlap

**Figure 4:**
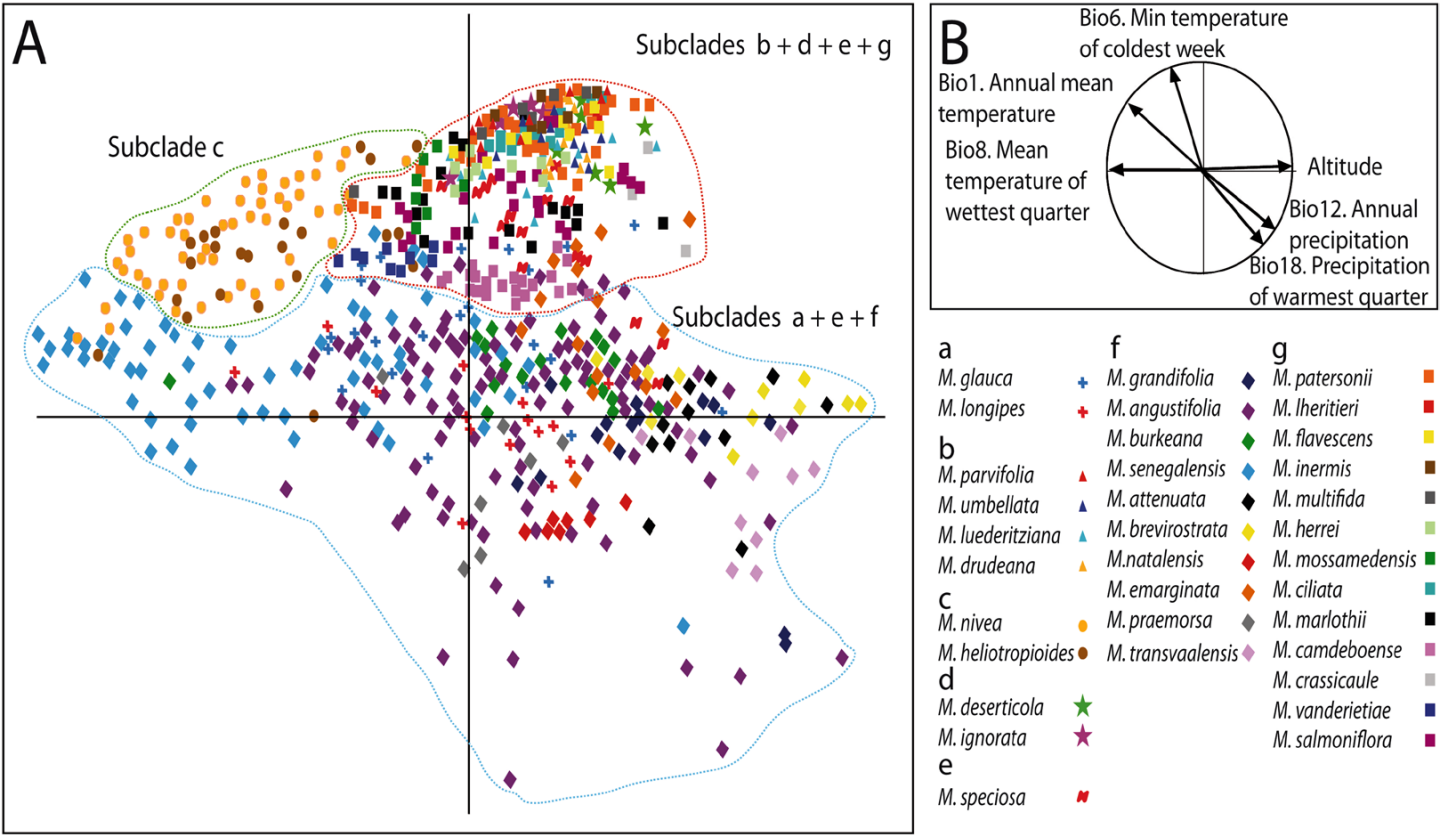
Niche analyses showing the results of the PCA of the 34 *Monsonia* species, each species labelled with a different symbol. The analysis was performed with nine variables that captured ca. 69% of the variance in the first two components (PC1: 41.1%, PC2: 28.0%). (**A**) PCA of all 34 *Monsonia* species. (**B**) Weight of the variables in the PCA analysis. Symbols are: a) *M*. gr. *longipes* (red and blue crosses), b) *M*. gr. *umbellata* (triangles), c) *M*. gr. *nivea* (yellow and brown spots), d) *M*. gr. *deserticola* (green and pink stars), e) *M*. *speciosa* (red N), f) *M*. gr. *attenuata* (diamonds), and g) *M*. gr. *Sarcocaulon* (squares).

**Figure 5:**
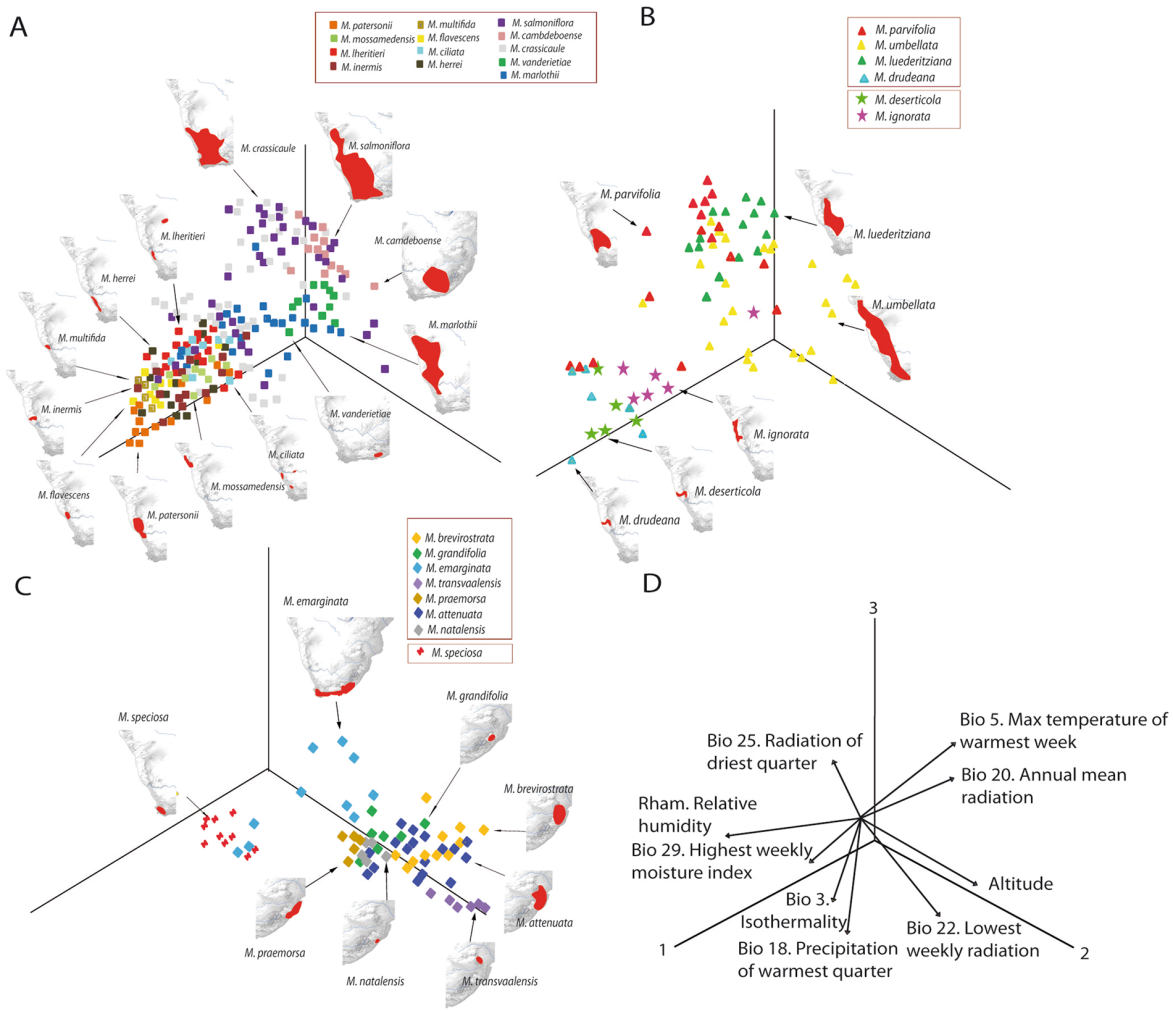
Niche analyses showing the results of the PCA for the dataset of South African species excluding widespread species; each species labelled with a different symbol. The analysis was performed with nine variables which captured ca. 83% of the variance in the first three components (PC1: 33.5%, PC2: 30.3%, PC3: 16.6%). (**A**) *Monsonia* subg. *Sarcocaulon* (subclade g in Fig. 2). (**B**) *Monsonia* gr. *umbellata* (triangles) and *M*. gr. *deserticola* (stars) (subclades b and d in Fig. 2). (**C**) *Monsonia* gr. *attenuata* (diamonds) and *M*. *speciosa* (red crosses) (subclades e and f in Fig. 2). (**D**) Weight of the variables in the PCA. Maps obtained with the software ArcGIS version 10.3.

To study the climate niche of *Monsonia* species, we obtained SDMs with high predictive accuracy, according to the AUC criterion (Area under the receiver operating curve). These distributions were consistent with their known distribution range of each of the 34 species analyzed (Fig. 4). Moreover, the Principal Component Analysis (PCA) of the complete *Monsonia* dataset (Fig. 4) captured c. 84.5% of the variance in the first three components (PC1: 41.1%, PC2: 28.0%, PC3: 15.4%) (Table S1) and divided *Monsonia* species along the first axis (PC1) into two groups: those occurring in warmer habitats, such as the northern African savannas and deserts, and those inhabiting cooler ecotypes, such as the Drakensberg Mountains and the Namib coastal belt (Fig. 4). The second axis PC2 divided the desert species from those growing in wetter environments. In total, three major groups were recovered: a group of species distributed in the Sahara Desert (subclade c), the group of Namib-Karoo species (subclades b, d, e, g), and a heterogeneous group formed by species occurring in savannas, steppes and grasslands (subclades a, e, and f). *Monsonia speciosa* (Cape region) appears in both the second and third groups, while *M*. *glauca*, *M*. *vanderietae* and *M*. *camdeboense* appear in intermediate positions between these groups (Fig. 5). Niche breadth values were calculated with the variables selected for the complete dataset analysis (Fig. 3; Table S2). *Monsonia nivea* exhibited the widest climatic niche (0.690), followed by *M*. *heliotropioides* (0.4933), *M*. *senegalensis* (0.4529), *M*. *burkeana* (0.4436), and *M*. *angustifolia* (0.354). The remaining species showed narrower niche breadths. The lowest values were found in Namib-Karoo coastal species such as *M*. *multifida* (0.0086), *M*. *inermis* (0.0149), *M*. *lheritieri* (0.0209), *M*. *drudeana* (0.0238) or *M*. *deserticola* (0.0299).

In order to increase the power of niche ordination and permit a good selection of variables for species exclusive to South African biomes, we also performed an analysis using a reduced dataset which included the species restricted to this region (e.g. 27 species). The PCA of this dataset (Fig. 5) captured c. 80.4% of the variance in the first three components (PC1: 33.5%, PC2: 30.3%, PC3: 16.6%; Table S3). This analysis also resulted in significant differentiation under the ANOSIM test (p < 0.01) and the average AUC. The test values of the nine-variables models for the studied lineages were >0.9. The PCA plot (Fig. 5) shows the species divided into three subplots: the first includes the species in subclade (g) (succulents); the second shows those in subclades (b) and (d) (desert herbs), while the third represents species included in subclade (f) (grassland herbs). The first axis was mostly correlated to Bio29 (highest weekly moisture index), Bio18 (precipitation of the warmest quarter) and relative humidity, and separated the species included in the Namib-Karoo-Cape group from those of the Natal-Drakensberg group. The second axis was significantly correlated to Bio18 (precipitation of the warmest quarter), and divided the Natal mountain species from the lowland ones. Finally, the third axis was correlated to Bio5 (maximal temperature of the warmest week), and discriminated between the coastal specialists and species growing in the coast and the inner parts of the Namib-Karoo-Cape within subclades (b), (d) and (g) (Fig. 5).

Pairwise comparisons of niche overlap among South African species are presented in Table 1. Values ranged from D = 0.009 (*M*. *vanderietiae* versus *M*. *deserticola*) to D = 0.832 (*M*. *parvifolia* versus *M*. *luederitziana*). In general, niche overlap among taxa was moderate to low, according to the classification of Rödder & Engler^26^, even between close relatives (D < 0.5). Moreover, niche-identity and niche-overlap tests showed that the highest overlap and similitude occurred between non-sister species included within subclades (g) and (b). In contrast, niche overlap among species for the remaining clades was marginal. The highest overlap values (D > 0.7) observed between two pairs of species belonging to the same subclade were D = 0.792 for *M*. *ciliata*-*M*. *herrei* (subclade g) and D = 0.832 for *M*. *parvifolia*-*M*. *luederitziana* (subclade b). In other cases, high niche overlap was observed between species belonging to different subclades, especially in species of the Namib Desert and Succulent Karoo coastal regions, for instance D = 0.723 between the herbaceous *M*. *drudeana* (subclade b) and the succulent *M*. *flavescens* (subclade g) (Table 1).

**Table 1:** Pairwise similarity in the ecological niche using the indexes D and I in selected sister and non-sister species and time distances among them (**divergent with a 99% signification).

Clade (s: sister species)	Species pair	Time distances (Ma)	Biomes of species 1	Biomes of species 2	I	D
g (s)	*M*. *herrei – M*. *multifida*	0.64	SK	SK	0.790**	0.498**
	*M*. *crassicaule – M*. *flavescens*	1.54	SK, NK, F	SK	0.628**	0.318**
	*M*. *salmoniflora – M*. *vanderietiae*	1.56	SK, NK, D	F	0.378**	0.149**
	*M*. *camdeboense – M*. *patersonii*	1.16	F, NK	SK, NK	0.264**	0.112**
g	*M*. *ciliata – M*. *herrei*	3.96	SK	SK	0.963	0.792
	*M*. *flavescens – M*. *inermis*	4.08	SK	SK	0.949	0.769
	*M*. *flavescens – M*. *multifida*	4.08	SK	SK	0.913	0.697
	*M*. *inermis*- *M*. *herrei*	2.84	SK	SK	0.846	0.637
	*M*. *inermis*- *M*. *multifida*	2.84	SK	SK	0.811**	0.547**
	*M*. *marlothii – M*. *mossamedensis*	8.66	SK, NK, D	D	0.791**	0.493**
	*M*. *ciliata – M*. *flavescens*	2.10	SK	SK	0.751	0.526
	*M*. *inermis*- *M*. *mossamedensis*	7.41	SK	D	0.620**	0.314**
	*M*. *inermis*- *M*. *marlothii*	6.89	SK	SK, NK, D	0.587**	0.288**
	*M*. *crassicaule*- *M*. *herrei*	4.08	SK, NK, F	SK	0.566**	0.300**
	*M*. *ciliata – M*. *crassicaule*	1.95	SK	SK, NK, F	0.497**	0.260**
	*M*. *marlothii – M*. *crassicaule*	8.66	SK, NK, F	SK, NK, D	0.469**	0.227**
	*M*. *lheritieri*-*M*. *salmoniflora*	6.20	SK	SK, NK, D, F	0.373**	0.156**
	*M*. *lheritieri*-*M*. *vanderietiae*	6.20	SK	F	0.094**	0.017**
f (s)	*M*. *natalensis – M*. *transvaalensis*	0.20	ND	ND	0.169**	0.071**
	*M*. *emarginata – M*. *praemorsa*	0.38	ND	ND	0.604**	0.349**
f	*M*. *grandifolia – M*. *praemorsa*	1.56	ND	ND	0.810**	0.521**
	*M*. *attenuata – M*. *brevirostrata*	6.89	ND	ND	0.900	0.658
b (s)	*M*. *luederitziana – M*. *umbellata*	3.32	SK, NK, D	SK, NK, D	0.845**	0.571**
b	*M*. *luederitziana – M*. *parvifolia*	6.60	SK, NK, D	SK, NK	0.974	0.832
d (s)	*M*. *deserticola – M*. *ignorata*	24.3	SK	SK	0.603**	0.321**
g-d	*M*. *deserticola – M*. *vanderietiae*	36.6	SK	F	0.052**	0.009**
	*M*. *deserticola – M*. *patersonii*	36.6	SK	SK, NK	0.844	0.583
g-b	*M*. *flavescens – M*. *drudeana*	43.6	SK	SK	0.940	0.723
	*M*. *salmoniflora – M*. *parvifolia*	43.6	SK	SK	0.852	0.608
	*M*. *salmoniflora – M*. *luederitziana*	43.6	SK, NK, D	SK	0.903	0.684
	*M*. *marlothii – M*. *umbellata*	43.6	SK, NK, D	SK, NK, D	0.880	0.664
	*M*. *inermis – M*. *drudeana*	43.6	SK	SK	0.890	0.629
	*M*. *patersonii – M*. *drudeana*	43.6	SK	SK	0.905	0.667
d-b	*M*. *drudeana – M*. *deserticola*	43.6	SK	SK	0.885	0.708

Biomes: SK, Succulent Karoo; NK, Nama Karoo; D, Namib desert; N, Natal-Drakensberg; F, Fynbos.

### Niche evolution

**Figure 6:**
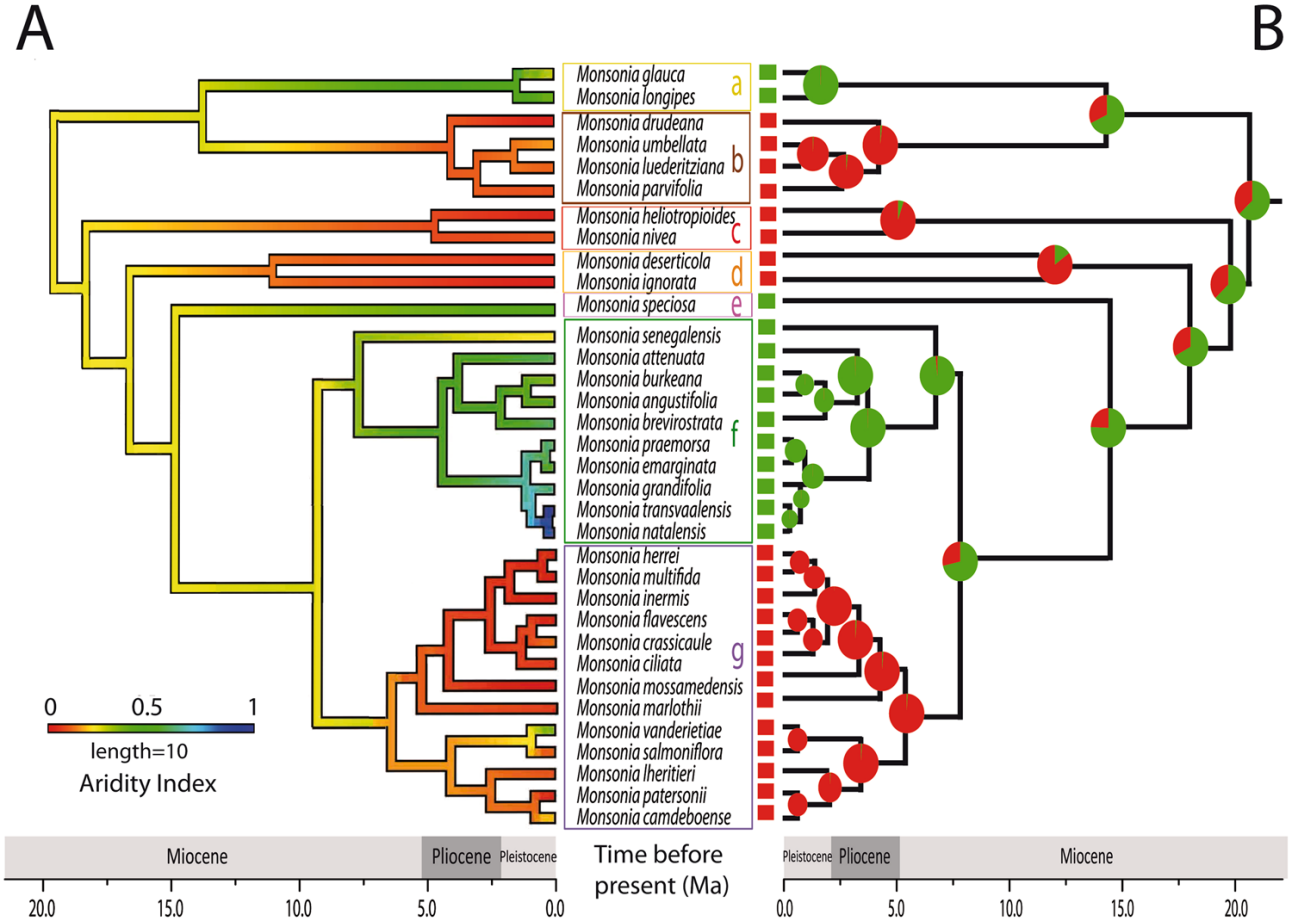
Niche and fruit trait evolution. (**A**) Shifts between arid and semiarid-wet habitats inferred using aridity values in the R package phytools with the Ornstein–Uhlenbeck continuous evolutionary model. (**B**) Reconstruction of fruit type evolution using Bayesian ancestral state inference in BayesTraits. Red: plumose (anemochorous); green: barbate (trypanocarpic). The phylogenetic reconstruction used is that of the MCC tree from the BEAST analyses.

Terminal and ancestral estimates of mean values of the aridity indexed – inferred using the (adaptive) Ornstein–Uhlenbeck (OU) continuous evolutionary model - are plotted on the MCC phylogenetic tree in Fig. 6, and for the other selected bioclimatic variables, humidity, precipitation, and temperature (Figs S4, S5 and S6). The reconstruction of niche occupancy through time revealed that the ancestral condition for the genus *Monsonia* was estimated with moderate aridity index values (Fig. 6). Evolution of climatic preferences towards high precipitation and humidity were found in subclades (a), (e), and (f), corresponding to the Great Rift, Cape and Natal-Drakensberg regions, while evolution to high aridity and low precipitations occurred in subclades (b), (c), (d), and (g) mostly distributed in the Succulent Karoo, the Namib and the Nama Karoo. Evolution to low temperatures was found in some species of subclade (f) (*M*. *attenuata*, *M*. *grandifolia*), and some Namib-Succulent Karoo coastal species (e.g., *M*. *drudeana*, *M*. *deserticola* and *M*. *ignorata*, Fig. S5); the opposite pattern was found in *M*. *senegalensis* and the two species of the Saharo-Arabian deserts (*M*. *nivea* and *M*. *heliotropioides*), which evolved preferently towards higher temperatures.

### Changes in niche preferences, habit type and dispersal mode

In terms of niche breadth, eleven anemochorous species of the Namib-Karoo show very narrow niches and smaller distributions. However, broad niches were  also observed: barbate species of subclade (c) (Saharo-Arabian deserts) and other within subclade (b) (Succulent Karoo and Nama Karoo) exhibit considerably larger niches. Though ancestral state reconstruction of fruit type evolution suggests that the ancestral fruit type in *Monsonia* is trypanocarpic, *Pelargonium*, the basal-most genus in the Geraniaceae, produce anemochorous fruits. When including outgroup choices, the analyses increases the probability of an anemochorous ancestor (data not shown). Our PGLS results suggest a significant correlation between aridity and fruit type (*p* < 0.001; Table S4): species that have plumose fruits occupy more arid habitats, while the opposite can be observed for trypanocarpic fruits. However, niche breadth and fruit type are not correlated (*p* = 0.1745).

Changes in habit or life form can be observed in three species of subclade (f), with evolution to succulent stems in all species of subclade (g). These changes were accompanied by shifts in the dispersal mode, often in the same direction: from anemochorous in arid or hyper-arid biomes to trypanocarpic in wetter ones and viceversa (Fig. 6).

### Photosynthetic metabolism: carbon isotope ratios (δ^13^C) in *Monsonia*

Carbon isotope ratio measurements for *Monsonia* range from −33.5‰ in *M*. *nivea* to −22.1‰ in *M*. *parvifolia* (Table S5), indicating that none of the species exhibits C_4_ or CAM photosynthesis with a high proportion of night assimilation^27^. This also confirms the results of Mooney *et al*.^28^, who described *M*. *mossamedensis* as a C_3_ species (values of −24.5‰ and −26.1‰). The ratio of −18.3‰ published by Rundel *et al*.^22^ is not comparable with ours, since it was calculated differently. Species studied in subclades (c) and (d) (herbaceous, Cape and Namib-Karoo) and subclade (g) (succulents, Cape and Namib-Karoo) had slightly lower values for this trait than the rest of species. Both leafy and succulent species, showed δ^13^C values significantly higher than those typical for CAM and C_4_ plants, which suggests a C_3_ photosynthetic pathway.

## Discussion

### Aridification, biome range expansions, and the origin of the African Arid Flora

Linder^2^ postulated that the AAF flora originated and diversified in Africa^2^, but that it also included some Australian and Eurasian elements. In his view, there are two arid Floras in Africa: one associated with south-west African coast and the Namib Desert (the “AAF”), and the other distributed in the eastern part of the continent, in the Somalia-Masai regional centre of endemism^2^. On the other hand, the “Arid Track”, also termed the African Arid Corridor (AAC), has been defined as a pattern connecting the floras of the southwest with the northeastern arid regions of Africa^9, 29, 30^. Bellstedt *et al*.^17^ defined the AAC pattern as the disjunction occurring between Southern Africa and Eastern African-Southern Arabian xeric floristic elements. This biogeographic pattern is related to another example of continental-wide disjunction, the Rand Flora^31, 32^. Linder^2^ considered the Rand Flora (RF) as an expansion of the AAC to the west. Some RF lineages include succulent plants, adapted to xeric environments (e.g., *Camptoloma*, *Euphorbia balsamifera*), though they never occur in deserts and they exhibit a wider disjunct distribution extending to northwest Africa and Macaronesia. Pokorny *et al*.^9^ considered the RF pattern as a pattern describing a more subtropical or sub-xeric, temperate ancient flora than the AAC. In any case, the origin of the RF and ACC patterns can probably be traced back to the aridification trend that affected the African continent from the Neogene onwards.

Paleoclimate records indicate that desertification in Africa started in the Namib Desert in the Early Miocene and progressed eastward and northwards^9, 11^. This temporal pattern fits well our results in *Monsonia*, with the most probably origin of the genus inferred in southwest Africa c. 21 Ma (Fig. 2), where subclade (b) is endemic, and subsequent expansions to the east and north in subclades (a), (c) and (f). *Monsonia* pollen was recorded offshore of Namibia in the cores retrieved at the Ocean Drilling Program (site 1081) in the Tortonian (7.5–9 Ma), but its abundance decreased in the Messinian, and even further during the Pliocene, probably as a consequence of climate aridification^15^. South western African species had thus a long-time to adapt to the increasingly arid conditions, and eventually settled in the foggy and cooler habitats close to the coasts. This could have triggered local speciation within a mosaic-like habitat structure which encloses fine-scale climatic gradients, and allow the accumulation of nearly sympatric species in the subclades (b), (d) and (g) (Fig. 2).

The relatively longer branches and lower speciation rates characterizing the basally diverging subclades (a-e) (Fig. 3) stand in contrast with the shift towards higher speciation rates inferred in the MRCA of subclades (f) and (g). It is possible that these early diverging clades represented older, less drought-tolerant lineages that scaped from the ongoing aridification by moving northeastward, for example subclade (a) (Fig. 2). The stem-age of *Monsonia* (32 Ma, CI 24.8–39.5) is in agreement with the Late Eocene-Early Oligocene Climate Cooling event^33^ (LEOC), which in some parts of Africa led to replacement of a subtropical flora by a more arid flora^10^ (Fig. 2). Since desertification during the Neogene advanced from the southwest to the north^11^, dispersal to the north and east was accompanied by adaptation to wetter habitats or biomes such as savannas, steppes, and wet forests, and sometimes by an increase in niche breadth (i.e. subclades a and c, Fig. 3). This is the case of the widespread (North African) species *M*. *nivea* and *M*. *heliotropioides* in subclade (c), but also of *M*. *senegalensis* in subclade (f), which has a range extending northwards into the grasslands and steppes of East Africa (Figs 2 and 3). The uplift of the Drakensberg Mountains in the Pliocene seems to have facilitated some of these these range expansions. Migration from the Drakensberg northwards has been reported in several plant groups^34^. In *Monsonia*, most species in subclade (f) are endemic to these mountains, but two of them (*M*. *angustifolia* and *M*. *senegalensis*) extend their ranges into the grasslands and steppes of southwest and east Africa. However, in a few cases, a migration to the south can be reported in subclades (f) and (g), such as in *M*. *emarginata* and *M*. *vanderietiae*, distributed in the Cape coasts.

### Ecological divergence and niche specialization explains diversification in *Monsonia*

The high diversification rates inferred for sister subclades (g) and (f), distributed in the southwest and southeast regions of Africa, seem to represent a young radiation driven by adaptive evolution (Fig. 3). Divergence from their sister-group, *M*. *speciosa*, was dated c. 14 Ma, after the Mid-Miocene Climate Optimum that initiated a global cooling trend^33^, and initial diversification within each subclade ranged from 6 to 4 Mya, following a general cooling trend in western South Africa and the rising of the Drakensberg Mountains in the East. Ancestral niche reconstruction suggests that the initial split between the two subclades (g) and (f) involved “ecological divergence” (niche evolution), the adaptation to different aridity niche values, as shown by the OU model (Fig. 6). Subclade (f) shows adaptation to wetter and colder habitats, whereas the succulent species in subclade (g) are adapted to more arid conditions (Fig. 2). The latter includes species of the two ecological groups: one comprises species of the Namib desert and Succulent Karoo coastal-belt, while the other is formed by species distributed in the coastal strip and the Nama-Karoo inner regions (Figs 2 and 6). Only few succulent species show niche overlap (Table 1), and none of them is reciprocal sister-species, suggesting that niche conservatism did not play a large role in the diversification of subclade (g). Indeed, this pattern of adaptation to different ecological optima is observed in other subclades in the phylogeny, such as in the divergence between subclade (b) (arid-adapted species) and subclade (a), in which range expansion to the east and north seem to have been accompanied by adaptation to semi-arid and wetter habitats (Figs 2 and 6).

On the other hand, the reduced niche exhibited by the coastal species in subclade (g) compared to those in other subclades (Fig. 3) is likely related to their specialization in the hyper-arid but cooler and foggy habitats present in these areas (Figs 5 and S6). The vegetation of the coastal belt of the Succulent Karoo and the Namib coast is largely dependent on lower temperatures and sea fog generated by the north-flowing Benguela current^13^. The difference in temperature between the cold sea and the warmer inland regions moves the fog mainland, towards the lower pressure points, which makes it condense on small rocks, outcrops and mountain slopes, where a large concentration of biodiversity has been reported^8, 35–37^. Genera such as *Conophytum* ^38^, *Portulacaria* ^39^, *Hoodia* ^40^, and *Pelargonium* ^41^ include taxonomic groups with levels of diversity in dry Western African habitats comparable to those in *Monsonia*.

*Monsonia* species exhibit two dispersal syndromes (wind and trypanocarpic) that have favoured the colonization of different types of habitats (Figs 6 and S7, Tables S6 and S7). These syndromes are associated with different dispersal abilities and related to distinct ecological niche patterns. Tripanocarpy is more common in semi-arid or wet habitats, permitting the seeds germinate near the parent plants, while anemochory is advantageous in arid habitats by favouring long distance dispersals^42^. Although BayesTraits reconstruction of the fruit type suggests that trypanocarpic is the ancestral state of *Monsonia *(PP = 0.630), this software attributes some probability to it being anemochorous (PP = 0.370). Contrarily, Maximum likelihood analyses in Mesquite, recovered the ancestral state as anemochorous, although with poor confidence. Anyway, our PGLS results clearly suggest a negative correlation between trypanocarpic fruits and the aridity index, with species exhibiting this type of fruit distributed in less arid habitats, like the Great Rift, the Cape, the Natal-Drakensberg and other wet or semiarid regions whereas plumose fruits are associated to species of the Namib-Karoo and the Sahara deserts (Tables S4, S6 and S7). Thus, these results reflect that species inhabiting arid environments prioritize the dispersal of the seed, while species occurring in wetter habitats focus on seedling survival. Consequently, changes in dispersal type enabled the colonization of novel biomes such as arid environments, which became dominant in the Late Miocene. To unravel the evolutionary history of the fruit type in *Monsonia*, further analyses with a broader sampling of the Geraniaceae will be required, which are far beyond the aims of this study.

Finally, the succulent life form seems to have evolved only once in the phylogeny (subclade g), likely as a consequence of the adaptation to arid environments in the Namib-Karoo Desert. Unlike other arid-adapted taxa with a CAM metabolism^43^ all succulent species of *Monsonia* analyzed here showed δ^13^C values, indicative of a C_3_ photosynthetic pathway. This type of photosynthesis has been reported in other southwestern African succulents such as Crassulaceae and Aizoaceae^44^. These autors suggest that the use of C_3_ photosynthesis interspersed with periods of no positive carbon assimilation may be a suitable strategy, alternative to CAM, in the southwestern African deserts.

## Conclusions

Africa represents a model continent to examine the long-term effects of past climatic changes, given the ongoing aridification trend that has affected the continent for the last 20 million years. Africa also harbours striking biogeographic patterns such as continent-wide disjunct distributions in plants, with sister clades or species separated sometimes by thousands of kilometers^9^ (this study). Whether these diversity and geographic patterns were formed by allopatric (neutral) speciation and extinction driven by abiotic factors, or by dispersal coupled with biotic adaptations (adaptive speciation, climate niche, morphology) is a crucial question to understand the causal mechanisms underlying species diversification in extreme environments. Our study reveals that a combination of these factors explains patterns in the species-rich, disjunctly-distributed *Monsonia*: high diversity is coupled with narrow niche breadths in species occurring in coastal arid-hyperarid regions of southwestern Africa, whereas species extending their ranges to other regions in Africa exhibit broader niches. Different fruit types (anemochorous and trypanocarpic) and life forms (annual, perennial and succulents) favoured the settlement to particular biomes.

## Methods

### Study group and taxon sampling

To clarify phylogenetic relationships within *Monsonia*, 34 species out of a total of 39 were sampled (Table S8), representing 87.2% of all species currently included in the genus. We included representatives of all other genera of Geraniaceae as outgroups, and *Hypseocharis* (Hypseocharitaceae) as a more distant outgroup^45–47^. We compiled an extensive dataset of distribution records, from the entire distributional range of *Monsonia* using data from literature, herbarium specimens, and our own fieldwork (Table S8).

### DNA sequencing

Total DNA was extracted from silica gel-dried plant tissue using the ‘DNeasy Plant Mini Kit’ (QIAGEN Inc., California, USA) according to manufacturer’s instructions. To reconstruct phylogenetic relationships within *Monsonia*, four variable cpDNA regions were sequenced: *trn*L-*trn*F, *trn*S-*trn*G, *mat*K5F-*C2M*R, and *rbc*L1F-*rbc*L724R producing a total of 107 new sequences (Table S8). We also sequenced the nuclear ribosomal marker ITS, producing a total of 11 new sequences. For details on PCR amplification and sequence alignment see Supplementary Information and Table S9. Sources of the material examined, location of vouchers, GenBank accession numbers, and full references are listed in Table S7.

### Phylogenetic dating, diversification analyses, and biogeographic inference

Phylogenetic relationships and divergence times were estimated with relaxed molecular clocks implemented in the software BEAST v. 1.7.5^25^. Based on the dating analyses of Sytsma *et al*.^47^, we used two secondary age constraints to calibrate: a) the split between *Hypseocharis bilobata* and the other taxa (i.e., the root node), and b) the split between *Pelargonium* and *Monsonia*. We used normal prior distributions representing the mean and 95% high posterior density (HPD) credibility intervals: (48 ± 2.5 Ma, and 36 ± 3.0 Ma, respectively) to account for the uncertainty in the original estimates. To identify shifts in diversification rate in the genus, we used BAMM v. 2.2.0 (Bayesian Analysis of Macroevolutionary Mixtures)^48^. We accounted for incomplete taxon sampling in our dataset by providing global sampling proportions detailing a general proportion of missing taxa of 0.1, and used a conservative 0.1 value for the Compound Process Prior. Our input tree was the MCC chronogram generated from BEAST. This method has been criticized recently for incorrect modelling of rate shifts at unobserved/extinct lineages and improper priors, so results should be taken with caution.

Spatio-temporal evolution was inferred using the Dispersal–Extinction–Cladogenesis model (DEC)^49^ implemented in the R package *BioGeoBEARS* ^50^. We defined four geographic areas as operational units: (1 + 2) Namib-Succulent Karoo plus Cape Floristic Region; (3) Natal-Drakensberg (southeastern Africa); (4) East Africa; (5) Sahara Desert + Arabian Peninsula (Fig. 2).

### Species distribution modelling, niche quantification and climatic niche evolution

Species distribution modelling (SDM) was performed to estimate the potential distributions of *Monsonia* under current climatic conditions. The occurrence dataset included 1118 localities of 34 species of *Monsonia*, compiled from herbarium specimens and our own collections (Table S8). In order to explore the bias that may be caused by the presence of point endemics beside widespread species with large niche breadths, we followed two approaches: 1) we first analysed the 1118 records covering the entire range of the 34 species, and second, and 2) we analysed only the 800 records of the 24 South African species. Then, we used 20 eco-climatological variables in 30 arc-seconds, which were clipped i) for the whole distribution area of *Monsonia*, or ii) for species distributed only in South Africa (the detailed data is shown in the supporting information).

To assess the role of niche conservatism versus niche evolution and determine the range breadths we tested niche similarity between all pairs of species using the metric *D* ^51^ as implemented in the program ENMtools v. 1.0^52^, and a measure derived from the Hellinger distance called I^53^. We included the identity test *I* to determine if the distribution models of two species differ in their niche by pooling the locality data for both species and sampling randomly from the pooled occurrences to create pseudoreplicate datasets (100 pseudoreplicates in our analyses) of equal size. The niche breadth was measured using the ‘inverse concentration’ metric in ENMTools.

We assessed the evolution of the species’ climatic variables through ancestral state reconstruction methods implemented in the R package *phytools* v. 0.3–93^54^. The MCC tree obtained from the concatenate dataset was used to study the evolution of the climatic niche with four variables: the annual mean temperature, the annual mean precipitation, the aridity index and the highest weekly moisture index. We chose these variables or climatic traits because they retained an important part of the variance in the principal component analyses (PCAs). We calculated the mean value for each of the four variables per species and conducted maximal likelihood (ML) ancestral state inference with four models of continuous trait evolution: BM (Brownian model, random drift), OU, (Ornstein–Uhlenbeck model, a selective-adaptive model), White Noise (lack of phylogenetic signal), and Early Burst (deceleration of BM variance). To infer the evolution of the occupation of habitat types, we also used the aridity index (aridity index = mean annual precipitation/mean annual potential evapotranspiration, data and methods available at: http://www.cgiar-csi.org/data/global-aridity-and-pet-database). Models were compared by using the corrected Akaike information Criterion (AIC_c_) for small sample sizes, as implemented in the R package *geiger* v. 2.0.6^55^. The Ornstein–Uhlenbeck (OU) model of evolution was selected as the best-fit model for all variables. We used then the function anc.ML in *phytools* to infer by Maximum Likelihod ancestral character states at each node in the phylogeny under the OU model, and the function cont.Map to plot these continuous character traits onto the phylogeny in *phytools* ^56^. Sometimes the selective optimum and the root state cannot be properly identified under the OU model. Therefore, we also inferred ancestral trait values under the BM model, which accounts for phylogenetic autocorrelation in ancestral trait values and compared with these inferences. In our case, BM and OU models produced similar results.

### Evolution of morphological traits

To infer the evolution of the dispersal type (discrete characters), we reconstructed the ancestral states using the Bayesian approach described by Pagel *et al*.^57^ and Pagel & Meade^58^, as implemented in BayesTraits v. 2.0^59^, on the posterior treesample of the BEAST analyses. We considered two states: plumose (anemochorous) and barbate (trypanocarpic) mericarps (Table S7 ). All representatives of *Monsonia* produce mericarps with hairs on the inner part of the awn: these present similar lengths over the awn in the “plumose” mericarps, but long in the lower part and short or absent in the upper part in the “barbate” mericarps^60^. Awns, mericarp bodies, and awn hairs were measured with a caliper using ten different mericarps per locality to determine whether seeds of these species could disperse by anemochory or not. Also, the buoyance ability of mericarps was tested following the Maddox & Carlquist^61^ method (see Supplementary Material and Methods). In the BayesTraits analyses, we assumed an exponential prior with the mean drawn from a uniform hyperprior on the interval 0 to 10. The MCMC run for 10^8^ generations, sampling every 1000th generation. The first 10^7^ generations were discarded as burn-in. The value of the *ratadev* parameter was set so that the MCMC acceptance rate was between 20 and 40%. Each analysis was conducted three times, and similar harmonic mean likelihoods indicated that the MCMC chain had converged.

To test whether there is a correlation between the niche aridity and the different types of dispersal syndromes of *Monsonia*, we performed the phylogenetic generalized least squares analyses^62^ (PGLS) the *gls* function and the corBrownian and corPagel correlations in the R packages *nlme* and *geiger* ^55^. PGLS regressions consider the phylogenetic structure in order to estimate the correlation between two or multiple traits. We implemented this analysis with the package *geiger* because it allows the possibility of choosing the evolution model (for instance BM or OU).

### Photosynthetic metabolism: carbon isotope ratios (δ^13^C)

To detect evidence for carbon concentrating mechanisms, we conducted a broad screening of carbon isotope ratios (δ^13^C) in *Monsonia*. The two major carbon fixing enzymes RuBP carboxylase and PEP carboxylase fractionate the stable isotopes of carbon, 12 and 13, differently^63^, resulting in ranges of to −22‰ to −33‰^64^ are typical of C_3_ species and −9‰ to −20‰ of C_4_ species and obligate CAM species^65^. Intermediate δ^13^C values (−18‰ to −22‰) might indicate a significant proportion CO_2_ fixation at night^65^ or simply long term stomatal closure^27^. The carbon isotope ratio of ca. 2 mg samples from adult leaves was measured using isotope ratio mass spectrometry^63^. Samples were analysed with a mass spectrometer at the Institut für Geowissenschaften, University Mainz, Germany. The δ^13^C values were calculated according to the method of Craig^66^ and the results expressed as δ^13^C with respect to the PDB standard.

## Electronic supplementary material

Supplementary Information
