# Supplementary material for: Opposite trends in the genus Monsonia (Geraniaceae): specialization in the African deserts and range expansions throughout eastern Africa
Source: Sci Rep. 2017 Aug 29;7:9872. doi: 10.1038/s41598-017-09834-6 (PMC5575343; doi:10.1038/s41598-017-09834-6)
Supplement: Supplementary file 1 — Supplementary Information [file 41598_2017_9834_MOESM1_ESM.doc]

**Supplementary Information**

**Opposite trends in the genus *Monsonia* (Geraniaceae): specialization in the African deserts and range expansions throughout eastern Africa.**

Sara García-Aloy*^1^, Isabel Sanmartín^2^, Gudrun Kadereit^3^, Daniel Vitales^1^, Ana María Millanes^4^, Cristina Roquet^5^, Pablo Vargas^2^, Marisa Alarcón ^1^† and Juan José Aldasoro^1,4^*†

**Supplementary Methods**

***DNA amplification, sequencing and alignment of DNA***

The four cpDNA regions and the nuclear ITS were amplified using the primers and settings shown in Table S9. DNA amplification was performed in an Eppendorf Master-cycler Epgradient S (Westbury, NY). Amplified products were treated with ExoSAP-IT (USB Corporation, Ohio) and submitted to Macrogen Inc. (Seoul, South Korea) and StarSeq (Mainz, Germany) for sequencing. The sequences of each region were aligned with MAFFT (Katoh *et al*., 2005) and checked by eye.

***Phylogenetic dating, diversification analyses, and biogeographic inference***

Phylogenetic relationships and divergence times were estimated with relaxed molecular clocks implemented in the software BEAST v. 1.7.5 (Drummond & Rambaut 2007), both on each independent molecular marker and on a concatenated dataset comprising all sequenced regions. To determine the best-fitting model of sequence evolution the Akaike Information Criteria (AIC) was used as implemented in Jmodeltest v. 2.2 (Posada, 2008), which resulted in a GTR + Γ for plastid markers and a GTR model for the ITS. We conducted four runs of 20^6^ generations each, sampling every 1000th generation. The resulting posterior distributions were checked using Tracer v. 1.4.1 (Drummond & Rambaut 2007). Four analyses were combined using LogCombiner v. 1.4.8, and the maximum credibility trees from the BEAST analysis were calculated using Tree Annotator v. 1.6, after removing a burn-in of 20%.

***Species distribution modelling and niche quantification***

Species distribution modelling (SDM) was performed using an occurrence dataset including 1118 localities of 34 species of *Monsonia*, compiled from herbarium specimens and our own collections. Geographic coordinates were obtained from GPS coordinates in the specimen labels or georeferenced localities. As input data for present climatic conditions, we used 19 eco-climatological variables in 30 arc-seconds downloaded from the WorldClim database (version 1.4 release 3; Hijmans *et al.*, 2005), and the average of relative humidity during the day (rham), which was obtained from Kriticos *et al.* (2012) (https://www.climond.org/). We followed two approaches to study the species climatic niche. First, 1) We performed a principal component analysis (PCA) for: a) 1118 records covering the entire distribution range of the 34 species analyzed, and b) 800 records from the 24 South African species. Thus, climate layers were clipped i) for the whole distribution area of *Monsonia*, and ii) for species distributed only in South Africa (longitude 10º to 36º; latitude -14º to -36º).

Second, we used the maximum entropy algorithm as implemented in MaxEnt v. 3.3 (Phillips *et al.*, 2006), to infer SDMs for all species; this method has been suggested as appropriate for low numbers of presence-only data, and its performance shown to be consistently competitive in comparison with other methods (Elith *et al.*, 2011). A first MaxEnt analysis on the entire dataset (comprising all 34 species) and the 20 bioclimatic variables produced a significant differentiation under the ANOSIM test (p < 0.01) and the average AUC (Area Under the receiver operating curve), the test values of the the nine-variables models for the studied lineages were above 0.9. We performed initial analyses on all variables and then chose nine climatic variables according to the MaxEnt models and those with a low coefficient of correlation (r < 0.7) (Table S1). Variables selected were: altitude, annual mean temperature (Bio1), maximal temperature of the warmest quarter (Bio5), minimal temperature of coldest week (Bio6), mean temperature of wettest quarter (Bio8), annual precipitation (Bio12), mean temperature of warmest quarter (Bio10), precipitation of driest quarter (Bio17), and precipitation of warmest quarter (Bio18). All MaxEnt analyses were performed using 10 replicates.

A second MaxEnt analysis was performed in a dataset including only the species distributed in South Africa to explore the bias in SDMs that might have caused the presence of widespread species with large niche breadths in the complete dataset (e.g., *Monsonia nivea*). In this dataset, MaxEnt selected nine variables out of the 20 bioclim variables: the average of relative humidity during the day (Rham), altitude (Alt), isothermality (Bio03), maximum temperature of warmest week (Bio05), precipitation of warmest quarter (Bio18), annual mean radiation (Bio20), lowest weekly radiation (Bio22), radiation of driest quarter (Bio25), and highest weekly moisture index (Bio29).

Niche breadth of each species was assessed by randomly sampling 100 pixels in the niche of each species (i.e. pixels were sampled according to the density of the species occurrences), extracting their scores along the two PCA axes, and calculating the standard deviation of the scores along the two PCA axes. This procedure was repeated 1000 times. Niche breadth is a metric with values ranging from 0 to 1 that represent the range of environments or resources a species can inhabit.

***Dispersal of fruits and seeds in Monsonia***

Two main diaspore types are easily identifiable in *Monsonia*: 1- anemochorous diaspores: plumose mericarps, with a loosely wound awn with long, feather-like hairs evenly distributed along the entire awn, and 2- autochorous and/or epizoochorous diaspores: barbate (trypanocarpic) mericarps with a spiral awn presenting bristly and durable persisting hairs on its basis which are absent or very short along the awn. Awns, mericarp bodies and hairs were measured with a caliper using ten different mericarps per species. To measure the ability of "lofting" (buoyancy in the air by a mericarp), we followed the procedure of Maddox & Carlquist (1985): a vertical tube of 1.5 m x 5 cm of transparent PVC was used with a fan at its base (2500 watts) which produced an airflow; air velocity increased and it was measured at the tube top until the mericarp stably floated towards the middle of the tube, and then the air speed was noted. The measurement was repeated 10 times with different ripe mericarps for each species (Table S7). Also, 50 mericarps were weighted using a precision balance.

**Supplementary Results**

Our experiments showed that barbate mericarps are less able to float in a stream of air (lofting), hence greater air velocity is needed for them to float (Table S7). The barbate mericarps present awns that twist in dried conditions in order to be buried in the ground (trypanocarpy), which prevents predation by harvester ants and other animals (Plitmann, 1986; Stamp, 1989; Evangelista et al., 2011; Abraham & Elbaum, 2013). Examples are the mericarps of *M. senegalensis*, which in the Kaokeveld are deposited by the ants in large quantities close to their nests (Moll et al., 2011). The barbate fruits may also adhere to the fur of animals and be transported by zoochory (e.g. by *Ovis* in *M. brevirostrata*, Ridley, 1990). In contrast, species that grow in arid or hyper arid zones tyipically present plumose mericarps. Most of them (but not all) are incapable of twisting in the ground and are not buried. However, plumose diaspores are also collected by ants, e.g. *M. umbellata* in the Namib-Karoo (Moll et al., 2011).

**Additional References**

Abraham, Y. & Elbaum, R. Hygroscopic movements in Geraniaceae: the structural variations that are responsible for coiling or bending. *New Phytologist*. **199**, 584–594 (2013).

Drummond, A.J. & Rambaut, A. BEAST: Bayesian evolutionary analysis by sampling trees. *BMC Evolutionary Biology*. **7**, 214 (2007).

Elith, J., Phillips, S.J., Hastie, T., Dudík, M., Chee, Y.E. & Yates, C.J. (2011). A statistical explanation of MaxEnt for ecologists. *Diversity and Distributions*. **17**, 43–57 (2011).

Evangelista, D., Hotton, S. & Dumais, J. The mechanics of explosive dispersal and self-burial in the seeds of the filaree, *Erodium cicutarium* (Geraniaceae). *The Journal of Experimental Biology*. **214**, 521–529 (2011).

Farquhar, G. D., Ehleringer, J.R., & Hubick, K.T. Carbon isotope discrimination and photosynthesis. *Annual review of plant biology.* **40**, 503-537 (1989).

Haberle, R. C. *et al.* Taxonomic and biogeographic implications of a phylogenetic analysis of the Campanulaceae based on three chloroplast genes. *Taxon*. **58**, 715-734 (2009).

Hamilton, M. B. Four primer pairs for the amplification of chloroplast intergenic regions with intraspecific variation. *Molecular Ecology*. **8** 521-522 (1999).

Hijmans, R. J., Cameron, S. E., Parra, J. L., Jones, P. G. & Jarvis, A. Very high resolution interpolated climate surfaces for global land areas. *International Journal of Climatology*. **25**, 1965–1978 (2005).

Johnson, L. A. & Soltis, D. E. Phylogenetic inference in Saxifragaceae sensu stricto and *Gilia* (Polemoniaceae) using matK sequences. *Annals of the Missouri Botanical Garden*. **82**, 149-175 (1995).

Katoh, K., Kuma, K., Toh, H. & Miyata, T. MAFFT version 5: improvement in accuracy of multiple sequence alignment. *Nucleic Acids Research*. **33**, 511–518 (2005).

Kriticos, D. J. *et al*. CliMond: global high resolution historical and future scenario climate surfaces for bioclimatic modelling. *Methods in Ecology and Evolution.* **3**, 53–64 (2012).

Maddox, J. C. & Carlquist, S. Wind dispersal in Californian desert plants: experimental studies and conceptual considerations. *Aliso: A Journal of Systematic and Evolutionary Botany*. **11**, 77–96 (1985).

Moll, E. e*t al.* Seed-collecting ants that run fairy rings around scientists or the mystery of the bare patches. *Veld & Flora*. **97**, 27–29 (2011).

Mooney, H.A., Troughton, J. H. & Berry, J.A. Carbon isotope ratio measurements of succulent plants in southern Africa. *Oecologia.* **30**, 295–305 (1977).

Olmstead, R. G., Michaels, H. J., Scott, K. M. & Palmer, J. D. Monophyly of the Asteridae and identification of their major lineages inferred from DNA sequences of *rbc*L. *Annals of the Missouri Botanical Garden*. **79**, 249-265 (1992).

Phillips, S.J., Anderson, R.P. & Schapire, R.E. Maximum entropy modeling of species geographic distributions. *Ecological Modelling.* **190**, 231–259 (2006).

Pierce, S., Winter, K. & Griffiths, H. Carbon isotope ratio and the extent of daily CAM use by Bromeliaceae. *New Phytologist.* **156**, 75–83 (2002).

Plitmann, U. Alternative modes in dispersal strategies, with an emphasis on herbaceous plants of the Middle East. *Proceedings of the Royal Society of Edinburgh*. *Section B. Biological Sciences*. **89**, 193–202 (1986).

Posada, D. jModelTest: phylogenetic model averaging. *Molecular Biology and Evolution.* **25**, 1253–1256 (2008).

Ridley H. N. The dispersal of plants throughout the world. *Koeltz Publishers Koenigstein, Germany* (1990).

Rundel, P. W., Esler, K. J. & Cowling, R.M. Ecoloical and phylogenetic patterns of carbon isotope discrimination in the winter-rainfall flora of the Richtersveld, South Africa. *Plant Ecology*. **142**, 133–148 (1999).

Stamp, N.E. Efficacy of explosive vs. hygroscopic seed dispersal by an annual grassland species. *American Journal of Botany*. **76**, 555–561 (1989).

Taberlet, P., Gielly, L., Pautou, G. & Bouvet, J. Universal primers for amplification of three non-coding regions of chloroplast DNA. *Plant molecular biology*. **17**, 1105-1109 (1991).

White, T.J., Bruns, T., Lee, S. & Taylor, J. Amplification and direct sequencing of fungal ribosomal RNA genes for phylogenetics. *PCR Protocols: A Guide to Methods and Applications* (ed. Academic Press, New York, 315–322, (1990).

**Tables**

**Table S1.** Weighting of each climatic variable in the PCA of all species. Values indicate the relative contributions of climatic variables to axis 1, 2 and 3.

| **Variable** | **Component 1** | **Component 2** | **Component 3** |
| --- | --- | --- | --- |
| Variance | 41.1% | 28.0% | 15.4% |
| Altitude | 0,2009214 | **-0,30858327** | **-0.54585253** |
| Bio1– Annual Mean Temperature | **-0.4801170** | -0.1982986 | 0.13454763 |
| Bio5 – Max Temperature of Warmest Month | **-0.4536112** | -0.11966219 | -0.23049589 |
| Bio6 – Min Temperature of Coldest Month | -0.2592365 | -0.09712167 | **0.62154019** |
| Bio8 – Mean Temperature of Wettest Quarter | -0.3303685 | -0.31192271 | -0.23871221 |
| Bio 10 – Mean Temperature of Warmest Quarter | -0.4855650 | -0.11567694 | -0.0996655 |
| Bio12 – Annual precipitation | 0.1743723 | -0.55833744 | 0.16496203 |
| Bio 17 – Precipitation of Driest Quarter | 0.2218058 | -0.35000629 | 0.38348203 |
| Bio18 – Precipitation of Warmest Quarter | 0.1778517 | **-0.54477547** | -0.05756475 |

**Table S2.** Niche analyses in *Monsonia*: number of localities, niche breadth, with uncertainty (UC), and mean annual precipitation for each species. The aridity index (AI) was used to classify the habitats between two major types: hyperarid or arid (a; AI < 0.2) and semiarid and relatively wet (s; AI > 0.2).

|  | **Species and subclade** | **Nº of localities** | **Niche breadth** | **UC** | **Mean annual precipitation (mm) (sd)** | **Aridity index**  **(sd)** | **Habitats**  **a / s** |
| --- | --- | --- | --- | --- | --- | --- | --- |
| a | *M. glauca*  *M. longipes* | 56  20 | 0.2750 | 0.9361 | 451 (229) | 0.276 (0.156) | s |
|  |  |  | 0.1875 | 0.9322 | 827 (343) | 0.545 (0.352) | s |
| b | *M. drudeana*  *M. luederitziana*  *M. parvifolia*  *M. umbellata* | 10 | 0.0238 | 0.7974 | 48 (26) | 0.040 (0.018) | a |
|  |  | 17 | 0.2098 | 0.9095 | 154 (57) | 0.090 (0.031) | a |
|  |  | 20 | 0.0898 | 0.8672 | 124 (41) | 0.071 (0.025) | a |
|  |  | 40 | 0.2981 | 0.9389 | 210 (113) | 0.121 (0.063) | a |
| c | *M. heliotropioides*  *M. nivea* | 26 | **0.4933** | 0.9736 | 128 (122) | 0.083 (0.080) | a |
|  |  | 23 | **0.6907** | 0.9850 | 60 (48) | 0.034 (0.026) | a |
| d | *M. deserticola*  *M. ignorata* | 12 | 0.0299 | 0.8143 | 80 (42) | 0.065 (0.033) | a |
|  |  | 13 | 0.0625 | 0.8598 | 45 (26) | 0.030 (0.040) | a |
| e | *M. speciosa* | 19 | 0.0716 | 0.8757 | 616 (283) | 0.474 (0.274) | s |
| f | *M. senegalensis*  *M. attenuata*  *M. burkeana*  *M. brevirostrata*  *M. angustifolia*  *M. grandifolia*  *M. transvaalensis*  *M. emarginata*  *M. natalensis*  *M. praemorsa* | 103 | **0.4529** | 0.9681 | 441 (289) | 0.243 (0.174) | s |
|  |  | 21 | 0.1277 | 0.9259 | 849 (179) | 0.624 (0.154) | s |
|  |  | 36 | **0.4436** | 0.9643 | 583 (177) | 0.370 (0.130) | s |
|  |  | 13 | 0.1887 | 0.9259 | 826 (183) | 0.639 (0.159) | s |
|  |  | 157 | **0.3545** | 0.9547 | 687 (325) | 0.426 (0.215) | s |
|  |  | 13 | 0.2184 | 0.9281 | 870 (127) | 0.654 (0.095) | s |
|  |  | 10 | 0.1696 | 0.9206 | 1060 (146) | 0.835 (0.138) | s |
|  |  | 22 | 0.0933 | 0.8830 | 639 (243) | 0.554 (0.095) | s |
|  |  | 6 | 0.0056 | 0.7029 | 1135 (10) | 0.989 (0.023) | s |
|  |  | 39 | 0.1975 | 0.9339 | 910 (151) | 0.675 (0.140) | s |
| g | *M. crassicaule* | 115  30  14  19  9  15  33  9  80  32  47  15  86 | 0.1740 | 0.9106 | 214 (172) | 0.151 (0.141) | a |
|  | *M. flavescens* |  | 0.0625 | 0.7144 | 61 (18) | 0.046 (0.015) | a |
|  | *M. ciliata* |  | 0.0953 | 0.8779 | 109 (29) | 0.081 (0.170) | a |
|  | *M. multifida* |  | 0.0086 | 0.7331 | 51 (9) | 0.039 (0.009) | a |
|  | *M. herrei* |  | 0.1286 | 0.8999 | 114 (40) | 0.079 (0.027) | a |
|  | *M. inermis* |  | 0.0149 | 0.7538 | 64 (19) | 0.047 (0.015) | a |
|  | *M. marlothii* |  | 0.1406 | 0.9037 | 149 (113) | 0.088 (0.064) | a |
|  | *M. mossamedensis* |  | 0.0226 | 0.7753 | 33 (22) | 0.021 (0.014) | a |
|  | *M. patersonii* |  | 0.0346 | 0.8244 | 61 (42) | 0.229 (0.049) | a |
|  | *M. camdeboense* |  | 0.0709 | 0.8583 | 337 (57) | 0.229 (0.049) | a |
|  | *M. lheritieri* |  | 0.0209 | 0.7926 | 172 (45) | 0.118 (0.033) | a |
|  | *M. vanderietiae* |  | 0.0490 | 0.8334 | 416 (64) | 0.391 (0.084) | s |
|  | *M. salmoniflora* |  | 0.2725 | 0.9347 | 163 (105) | 0.108 (0.084) | a |
|  | Total | 1118 |  |  |  |  |  |

**Table S3.** Weighting of each climatic variable in the PCA of South African species. Values indicate the relative contributions of climatic variables to axis 1, 2 and 3.

| **Variable** | **Component 1** | **Component 2** | **Component 3** |
| --- | --- | --- | --- |
| Variance | 33.5% | 30.3% | 16.6% |
| Altitude | -0.04141628 | **0.52449145** | -0.092067326 |
| Rham – Relative humidity during the daytime | **0.34799230** | **-0.46176563** | -0.188393798 |
| Bio 3 – Isotermality | -0.02349262 | -0.14278512 | **-0.648446682** |
| Bio 5 – Max Temperature of Warmest Month | -0.22193288 | 0.27008238 | **0.372748411** |
| Bio 18 – Precipitation of Warmest Quarter | **0.42687053** | **0.36630343** | -0.159216769 |
| Bio 20 – Anual Mean Radiation | **-0.54478218** | 0.06217355 | -0.003911671 |
| Bio 22 – Lowest Weekly Radiation | -0.26268875 | 0.28987012 | **-0.594671287** |
| Bio 29 – Weekly Moisture Index | **0.46888756** | 0.20710226 | 0.116204672 |

**Table S4. Results from model averaging and variable selection procedure with PGLS models. The values given in the table are the estimated coefficients of continuous variables (niche breadth, aridity index), and discrete variables (fruit type and life form), and corresponding p-values, correlation (R), AIC and as parameter estimate: alpha(a) or lambda (l) values.**

| Variables | Coefficient | p-value | Correlation (R) | AIC | alpha (a) or lambda (l) |
| --- | --- | --- | --- | --- | --- |
| Niche breadth - Aridity | -0.339738 | **0.0130** | -0.448 | -22.439 | a = 0.18715 |
| Niche breadth – Fruit plumose | -0.081610 | 0.1745 | -0.799 | -22.171 | a = 4.18464 |
| Aridity - Fruit plumose | -0.439556 | **0** | 0.801 | -31.537 | a = 2.64061 |

**Table S5.** Carbon isotope ratios (δ^13^C) measured in *Monsonia.* Values of -22‰ to -33‰ are typical of C_3_ species and values between -9‰ to -18‰ are typical of C_4_ species and obligate CAM species (Farquhar *et al.*, 1989; Pierce *et al.*, 2002).

| Species | Locality and collector number or reference | Average calibrated  N=3 | Stdev (batches) |
| --- | --- | --- | --- |
| *Monsonia angustifolia* | South Africa, N Transvaal; Penther 2218 (W) | -30.27 | 0.14 |
| *M. attenuata* | South Africa, Cathedral Peak; Carbutt 271 (MSUN) | -27.53 | 0.07 |
| *M.* *brevirostrata* | Lesotho, Qacha's Nek; Albers 4015b (MSUN) | -28.82 | 0.05 |
| *M.* *crassicaule* | Rundel et al.,1999 | -18.3* | * |
| *M.* *deserticola* | Namibia, Aus-Namib; Rand 100123 (MSUN) | -26.09 | 0.1 |
| *M. emarginata* | South Africa, Stutterheim, Pine Forest; Carbutt 312 (MSUN) | -31.78 | 0.06 |
| *M.* *grandiflora* | South Africa,10km NE Ixopo Highland Sourveld (MSUN) | -29.62 | 0.02 |
| *M. heliotropioides* | Algeria, Oudja; Molero BC-804188 | -29.17 | 0.13 |
| *M. ignorata* | Namibia, Windhoek, Sossusvlei; Vargas 421PV02 (MA) | -26.84 | 0.04 |
| *M. inermis* | Namibia, 3km SW Rosh Pinah (MSUN) | -28.59 | 0.03 |
| *M.* *lheritieri* | South Africa, Garies-Bitterfontein; Doring Krad A9078 | -24.2 | 0.01 |
| *M.* *luederitziana* | South Africa, 18 Km Fish River, Grünau; Klein, A9074 | -25.87 | 0.01 |
| *M. marlothii* | Namibia, Swakopmund, Khan canyon; Vargas 423PV03 (MA) | -27.94 | 0.05 |
| *M.* *mossamedensis* | Mooney *et al*., 1977 | -24.5, -26.1 | - |
| *M.* *natalensis* | South Africa, Port Edward; Umtauna 280CC | -30.21 | 0.03 |
| *M. nivea* | Morocco, High Atlas, S of the M'Goun Mt.; Staudinger s.n. | -33.45 | 0.02 |
| *M. parvifolia* | Namibia, Gariep, Great Buschmansland; Schlechter s.n. (W) | -22.12 | 0.03 |
| *M. patersonii* | Namibia, Aus-Witput A9054 (MA) | -27.85 | 0.06 |
| *M.* *salmoniflora* | Namibia, Aus-Witput 40km from Aus, 9049 | -25.85 | 0.05 |
| *M.* *senegalensis* | Ethiopia, Negele to Moyale, A10299 | -28.56 | 0.08 |
| *M. speciosa* | South Africa | -30.00 | 0.07 |
| *M.* *umbellata* | Namibia, cult. in Kirstenbosch; van Jaarsveld 8699 | -25.77 | 0.02 |

* Estimated in another study with a different method, here these values indicate C_3_ photosynthesis.

**Table S6.** Morphological and habitat features for *Monsonia* species studied here

| **Subclade** | **Species** | **Roots** | **Habit** | **Fruits** | **Biome** | **Habitat** |
| --- | --- | --- | --- | --- | --- | --- |
| **a** | *M. glauca*  *M. longipes* | Rhizome with tubers | Perennial | Barbate |  | Grasslands, steppes and savannas, 300-1800m |
|  |  | Rhizome with tubers | Perennial | Barbate |  | Grasslands, steppes and savannas, 600-3400m |
| **b** | *M. drudeana*  *M. luederitziana*  *M. parvifolia*  *M. umbellata* | Rhizome | Perennial | Plumose |  | Sands, riversides, 0-1000m |
|  |  | Herbaceous | Perennial | Plumose |  | Sands, pebble flats and riversides, 200-1400m |
|  |  | Rhizome | Perennial | Plumose |  | Sands, stony soils, pebble flats and riverbeds, 100-1500m |
|  |  | Rhizome | Perennial | Plumose |  | Sands, stony soils, and riverbeds, 0-1800m |
| **c** | *M. heliotropioides*  *M. nivea* | Rhizome | Perennial | Plumose |  | Sands, stony soils, cultivate places and riverbeds, 0-2200m |
|  |  | Rhizome, often with tubers | Perennial | Plumose |  | Sands, stony soils, and riverbeds, 0-2400m |
| **d** | *M. deserticola*  *M. ignorata* | Rhizome | Perennial | Plumose |  | Sands, gravelly, pebble flats, 0-1500m |
|  |  | Rhizome with tubers | Perennial | Plumose |  | Sands, 0-1400m |
| **e** | *M. speciosa* | Rhizome with tubers | Perennial | Barbate |  | Grasslands on sandy soils, 0-500m |
| **f** | *M. senegalensis*  *M. attenuata*  *M. burkeana*  *M. brevirostrata*  *M. angustifolia*  *M. grandifolia*  *M. transvaalensis*  *M. emarginata*  *M. natalensis*  *M. praemorsa* | Herbaceous root | Annual | Barbate |  | Disturbed sites, grasslands, steppes and savannas, 0-2600m |
|  |  | Rhizome with tubers | Perennial | Barbate |  | Grasslands and eroded sites among rocks, 1300-2400m |
|  |  | Rhizome | Perennial | Barbate |  | Disturbed sites, grasslands, steppes and savannas, 800-2000m |
|  |  | Herbaceous root | Annual | Barbate |  | Grasslands and eroded sites among rocks, 1500-2800m |
|  |  | Herbaceous root | Annual | Barbate |  | Disturbed sites, grasslands, steppes, and savannas 0-2200m |
|  |  | Rhizome | Perennial | Barbate |  | Grasslands and eroded sites among rocks, 300-1600m |
|  |  | Rhizome | Perennial | Barbate |  | Grasslands, 1000-2300m |
|  |  | Rhizome with tubers | Perennial | Barbate |  | Grasslands, steppes and savannas, 0-1100m |
|  |  | Rhizome with tubers | Perennial | Barbate |  | Grasslands, 0-600m |
|  |  | Rhizome with tubers | Perennial | Barbate |  | Grasslands, 0-700m |
| **g** | *M. crassicaule* | Shallow roots, not swollen | Perennial | Plumose |  | Stony soils and pebble flats, outcrops of quartzite, 0-1700 m |
|  | *M. flavescens* | Shallow roots, not swollen | Perennial | Plumose |  | Sandy or rocky flats, 0-1200 m |
|  | *M. ciliata* | Shallow roots, not swollen | Perennial | Plumose |  | Sandy or rocky flats, 100-600 m |
|  | *M. multifida* | Shallow roots, swollen | Perennial | Plumose |  | Stony soils and rocky flats, 0-600 m |
|  | *M. herrei* | Shallow roots, not swollen | Perennial | Plumose |  | Mountain sides, stony soils, outcrops of quartzite, 200-1000 m |
|  | *M. inermis* | Shallow roots, not swollen | Perennial | Plumose |  | Sandy or rocky flats, 0-1300 m |
|  | *M. marlothii* | Shallow roots, not swollen | Perennial | Plumose |  | Sandy or rocky flats, 0-1500 m |
|  | *M. mossamedensis* | Shallow roots, not swollen | Perennial | Plumose |  | Sandy or rocky flats, 0-800 m |
|  | *M. patersonii* | Shallow roots, not swollen | Perennial | Plumose |  | Sandy or rocky flats, 0-1700 m |
|  | *M. camdeboense* | Shallow roots, swollen | Perennial | Plumose |  | Mountain sides, rocky hills, 100-1600m |
|  | *M. lheritieri* | Shallow roots, not swollen | Perennial | Plumose |  | Sandy or rocky flats, 0-1200 m |
|  | *M. vanderietiae* | Shallow roots, not swollen | Perennial | Plumose |  | Mountain sides, rocky hills, outcrops of quartzite, 0-700 m |
|  | *M. salmoniflora* | Shallow roots, not swollen | Perennial | Plumose |  | Sandy or rocky flats, 0-1600 m |

**Table S7**. Mericarp features in *Monsonia*.

|  | Lofting speed mean in m/s (s.d.) | Mericarp length (body plus awn) in mm (s.d.) | Mericarp body length in mm (s.d.) | Mericarp weight in mg. (s.d.) | Mericarp type: plumose (p) or barbate (b) |
| --- | --- | --- | --- | --- | --- |
| *M. glauca* | **3.325 (0.05)** | 57.0 (7.1) | 13.5 (2.1) | 20 (3.2) | b |
| *M. longipes* | **5.0 (0.24)** | 63.1 (7.4) | 11.5 (3.2) | 14.1 (1.9) | b |
| *M. drudeana* | 1.41 (0.2) | 19 (1.2) | 5.9 (1.2) | 9.2 (1.2) | p |
| *M. parvifolia* | 1.475 (0.1) | 58 (6.2) | 6.2 (1.4) | 4.3 (1.1) | p |
| *M. luederitziana* | 2.075 (0.17) | 60 (7.0) | 5.6 (1.2) | 12.1 (3.2) | p |
| *M. umbellata* | 1.275 (0.1) | 61 (6.3) | 5.3 (0.87) | 4.6 (1.0) | p |
| *M. heliotropioides* | 0.862 (0.05) | 38 (2.2) | 5.2 (1.23) | 2.8 (0.8) | p |
| *M. nivea* | 0.838 (0.05) | 61 (6.2) | 5.1 (1.40) | 2.5 (0.0) | p |
| *M. ignorata* | 1.30 (0.08) | 17 (1.0) | 5.2 (0.81) | 10.4 (2.0) | p |
| *M. deserticola* | 1.57(0.05) | 37 (4.1) | 4.9 (1.1) | 2.0 (0.6) | p |
| *M. attenuata* | **3.51 (0.19)** | 48.0 (4.2) | 9.6 (2.5) | 11.8 (3.0) | b |
| *M. brevirostrata* | **2.77 (0.18)** | 16.4 (1.9) | 5.9 (1.9) | 2.4 (0.6) | b |
| *M. natalensis* | **3.75 (0.20)** | 57.0 (2.5) | 17.0 (3.2) | 19.5 (4.1) | b |
| *M. transvaalensis* | **3.80 (0.21)** | 6.10 (1.1) | 15.2 (3.0) | 17.5 (3.4) | b |
| *M. angustifolia* | **3.10 (0.18)** | 48.3 (5.3) | 7.6 (1.2) | 3.8 (0.9) | b |
| *M. senegalensis* | **3.625 (0.15)** | 45.0 (3.3) | 7.2 (0.9) | 8.0 (1.2) | b |
| *M. burkeana* | **3.089 (0.13)** | 63.0 (5.2) | 12.3 (4.2) | 7.3 (0.9) | b |
| *M. grandifolia* | **3.10 (0.12)** | 63.8 (5.9) | 12.5 (3.2) | 7.0 (1.0) | b |
| *M. lanuginosa* | **3.99 (0.21)** | 58.9 (4.1) | 12.5 (1.3) | 11.0 (1.2) | b |
| *M. emarginata* | **2.875 (0.17)** | 50.0 (4.2) | 13.5 (2.1) | 12.2 (1.5) | b |
| *M. praemorsa* | **4.10 (0.08)** | 48.0 (3.2) | 14.0 (2.0) | 18.5 (1.4) | b |
| *M. speciosa* | **4.475 (0.1)** | 66.0 (4.6) | 13.5 (1.4) | 20.0 (4.6) | b |
| *M. lheritieri* | 2.15 (0.13) | 65.0 (3.8) | 14.0 (2.2) | 25.4 (4.0) | p |
| *M. patersonii* | 2.05 (0.06) | 56.0 (4.2) | 5.3 (1.1) | 4.0 (0.7) | p |
| *M. salmoniflora* | 1.675 (0.1) | 39.6 (3.2) | 8.9 (1.2) | 5.1 (0.8) | p |
| *M. camdeboense* | 2.075 (0.1) | 48.0 (3.9) | 6.8 (1.2) | 2.8 (1.0) | p |
| *M. mossamedensis* | 2.50 (0.06) | 42.8 (4.0) | 7.0 (1.6) | 3.1 (0.6) | p |
| *M. crassicaule* | 2.825 (0.1) | 59.0 (6.2) | 13.0 (3.3) | 6.8 (1.7) | p |
| *M. marlothii* | 1.60 (0.1) | 43.0 (5.2) | 8.2 (1.3) | 4.0 (0.4) | p |
| *M. multifida* | 2.30 (0.08) | 40.0 (3.8) | 11.0 (2.3) | 7.0 (1.2) | p |
| *M. inermis* | 1.42 (0.11) | 38.2 (3.2) | 6.0 (1.4) | 4.9 (1.0) | p |
| *M. ciliata* | 1.67(0.12) | 43.1 (5.0) | 9.0 (1.2) | 4.7 (0.8) | p |
| *M. herrei* | 2.112 (0.21) | 48.0 (4.2) | 10.4 (2.3) | 5.4 (0.7) | p |
| *M. vanderietiae* | 1.175 (0.11) | 58.0 (6.1) | 11.4 (2.2) | 6.4 (1.1) | p |
| *M. flavescens* | 1.904 (0.15) | 54.4 (5.2) | 9.2 (2.0) | 4.0 (0.9) | p |

**Table S8.** Localities of the sampled populations of *Monsonia* and GenBank accession numbers. Numbers in bold indicate the new sequences, * indicate chloroplast complete-genome sequenced.

| Species | Locality and collector number | trnL-F | trnS-G | matK | rbcL | ITS |
| --- | --- | --- | --- | --- | --- | --- |
| *California macrophylla* | --- | DQ072015 | --- | --- | --- | EF185338 |
|  | --- | --- | JQ031013* | --- | --- | --- |
|  | --- | --- | --- | KJ916367 | KJ916488 | --- |
| *Erodium chrysanthum* | --- | KJ701602* | KJ701602* | KJ701602* | KJ701602* | --- |
|  | --- | --- | --- | --- | --- | EF185361 |
| *Erodium glandulosum* | Spain, Barcelona, Montcau ; Sáez 5001 | HQ677619 | **KX661374** | **KY630145** | **KX661375** | EF185367 |
| *Erodium foetidum* | --- | KF771022* | KF771022* | KF771022* | KF771022* | --- |
|  | Spain, Girona, Cap Norfeu; Aedo CA4920 | --- | --- | ------ |  | HQ677604 |
| *Geranium macrorrhizum* | --- | --- | --- | EU922243 | EU922281 | --- |
|  | France, Pyrenees; Aldasoro s.n. | **KX661378** | **KY630153** | --- | --- | DQ525073 |
| *Geranium nepalense* | --- | --- | -- | JF953873 | JF941756 | DQ192637 |
|  | Japan, Honshu; Estebanez, s.n. | **KX661376** | --- | --- | --- | --- |
| *Geranium nodosum* | --- |  | -- | KP963400 | JX913480 | --- |
|  | Italy, Abruzzo, Ceppo | DQ452922 | --- | --- | --- | **KX661379** |
| *Geranium platypetalum* | Turkey, Rize, Ovit Dağı Geçidi - Nisa 891 (MA) | **KX661377** | --- | --- | --- | AJ884938 |
|  | --- | --- | -- | KP963402 | KP963382 |  |
| *Hypseocharis bilobata* | --- | KF240616* | KF240616* | KF240616* | KF240616* |  |
|  | --- |  |  |  |  | HE795067 |
| *Hypseocharis pimpinellifolia* | --- | HE795472 | -- | -- | --- | HE795068 |
|  | --- | --- | --- | --- | DQ317048 | --- |
| *Monsonia angustifolia* | Ethiopia, Moyale to Mega; Aldasoro 10238 (MA) | DQ452891 | **KX470525** | **KX470466** | **KX470490** | --- |
|  | --- | --- | --- | --- | --- | AF505632 |
| *Monsonia attenuata* | South Africa, Cathedral Peak; Carbutt 271 (MSUN) | --- | **KY630154** | **KY630146** | **KX470491** | --- |
|  | --- | AY036161 | --- | -- | --- | AF505630 |
| *Monsonia brevirostrata* | South Africa, Lesobeng; Germishuizen 1238 (MO) | --- | **KX470526** | **KX470467** | **KX470492** | --- |
|  | --- | AY036159 | --- | --- | --- | AF505631 |
| *Monsonia burkeana* | Waterberg, Ratelhoek; Germishuizen 957 (PRET) | **KY576063** | **KX470527** | **KX470468** | **KX470493** | **KX470455** |
| *Monsonia camdeboense* | South Africa, cultivated in Kirstenbosch | DQ452900 | **KX470528** | **KX470469** | **KX470494** | **KX470456** |
| *Monsonia* *ciliata* | South Africa, Steinkopf, Anenous Pass; Aldasoro 9034 (MA) | --- | **KX470529** | **KX470470** | **KX470495** | **KX470457** |
|  | --- | AY036166 | --- | --- | --- | --- |
| *Monsonia* *crassicaule* | South Africa, 33 km Steinkopf; Aldasoro 2416 (MA) | --- | **KX470530** | **KX470471** | **KX470496** | --- |
|  | --- | AY036165 | --- | --- | --- | AF505637 |
| *Monsonia* *deserticola* | Namibia, Aus; Lavranos & Pehlemann 19636 (MO) | --- | **KY630155** | **KX470472** | **KX470497** | --- |
|  | --- | AY036178 | --- | --- | --- | AF505652 |
| *Monsonia* *drudeana* | Namibia, Gariep 13 km from the station; Aldasoro 9068 (MA) | **KX470521** | **KX470531** | **KX470473** | **KX470498** | **KX470458** |
| *Monsonia emarginata* | --- | --- | --- | --- | --- | HE795069 |
|  | South Africa, East London; Phyllipson 379 (UPS) | DQ452893 | **KX470532** | **KX470474** | **KX470499** | --- |
| *Monsonia* *flavescens* | South Africa, Oranjemund; Aldasoro 9099 (MA) | --- | **KX470533** | **KX470475** | **KX470500** | --- |
|  | --- | AY036164 | --- | --- | --- | AF505636 |
| *Monsonia* *glauca* | --- | AF505643 | --- | --- | --- | AF551333 |
|  | South Africa, Upington Karoides; UM581 (MSUN) | --- | **KY630157** | **KX470476** | **KX470493** | --- |
| *Monsonia* *grandifolia* | --- | AY036158 | --- | --- | --- | AF505629 |
|  | South Africa,10km NE Ixopo Highland Sourveld (MSUN) | --- | **KX470534** | **KX470477** | **KX470502** | --- |
| *Monsonia heliotropioides* | --- | AY036177 | --- | --- | --- | AF505651 |
|  | Algeria, Hoggar, Assekrem Mt. 1700 m; Aldasoro 9826 | --- | **KX470535** | **KX470478** | **KX470503** | --- |
| *Monsonia* *herrei* | South Africa, Steinkopf-Vioolsdrift; Aldasoro 9042 | **KX470522** | **KX470536** | **KX470479** | **KX470504** | **KX470459** |
| *Monsonia ignorata* | Namibia, Windhoek, Sossusvlei; Vargas 421PV02 (MA) | DQ072010 | **KY630156** | **KX470480** | **KX470505** | --- |
|  | --- | --- | --- | --- | --- | AF505647 |
| *Monsonia inermis* | Namibia, 3km SW Rosh Pinah (MSUN) | --- | **KY630158** | **KY630147** | **KX470506** | --- |
|  | --- | AY036162 | --- | --- | --- | AF505634 |
| *Monsonia* *lheritieri* | South Africa, Garies-Bitterfontein; Krad A9078 | **KX470523** | **KX470537** | **KY630148** | **KX470507** | **KX470460** |
| *Monsonia* *longipes* | Ethiopia, Negele 37 km to Moyale; Aldasoro10288 | **KX470524** | **KX470538** | **KX470481** | **KX470508** | **KX470461** |
| *Monsonia* *luederitziana* | --- | AY036173 | --- | --- | --- | AF505646 |
|  | South Africa, 18 Km Fish River Canyon; Aldasoro 9074 | --- | **KX470539** | **KX470482** | **KX470509** | --- |
| *Monsonia marlothii* | --- | --- | --- | KT692739* | --- | --- |
|  | Namibia, Swakopmund, Khan canyon; Vargas 423PV03 | DQ452899 | **KX470540** | --- | DQ452867 | KX470462 |
| *Monsonia* *mossamedensis* | --- | AY036169 | --- | --- | --- | AF505641 |
|  | Angola, S. Orumpembe, Skeleton Coast; 3603 (COI) | --- | **KY630159** | **KY630149** | **KX470510** | --- |
| *Monsonia multifida* | --- | --- | --- | --- | --- | AF505635 |
|  | South Africa, Kirstenbosch; Gassner 77 (MA) | DQ452898 | **KX470541** | **KX470483** | **KX470511** | --- |
| *Monsonia* *natalensis* | South Africa, Port Edward; Umtauna 280CC | --- | **KX470542** | **KX470484** | **KX470512** | --- |
|  | --- | AY036156 | --- | --- | --- | AF505626 |
| *Monsonia nivea* | Morocco, Col du Mekmek; Aldasoro 188865 | DQ452895 | **KX470543** | **KX470485** | **KX470513** |  |
|  | --- | --- | --- | --- | --- | AF505649 |
| *Monsonia parvifolia* | --- | --- | --- | --- | --- | AF505644 |
|  | South Africa, Vioolsdrift; Fairall s.n. (MO) | DQ452894 | **KX470544** | **KX470486** | **KX470514** | --- |
| *Monsonia patersonii* | Namibia, Aus-Witput; Aldasoro 9054 (MA) | --- | **KX470545** | **KX470487** | **KX470515** | **KX470463** |
|  | --- | AY036168 | --- | --- | --- | --- |
| *Monsonia praemorsa* | South Africa, Natal, Clermont; Wood s.n. (G) | DQ452892 | **KX470546** | **KY630150** | **KX470516** | **KX470464** |
| *Monsonia* *salmoniflora* | Namibia, Aus-Witput 46 km of Aus; Aldasoro 9049 | --- | **KX470547** | **KY630151** | **KX470517** | --- |
|  | --- | AY036167 | --- | --- | --- | AF505639 |
| *Monsonia* *senegalensis* | Ethiopia, Negele to Moyale; Aldasoro 10299 | --- | **KX470548** | **KY630152** | **KX470518** | --- |
|  | --- | AY036179 | --- | --- | --- | AF505633 |
| *Monsonia speciosa* | South Africa, Tulbagh Cape Colony | DQ452896 | **KX470549** | EU922387 | **KX470519** | --- |
|  | --- | --- | --- | --- | --- | AF505648 |
| *Monsonia* *umbellata* | Namibia, cult. in Kirstenbosch; van Jaarsveld 8699 , | --- | **KY630160** | **KX470489** | **KX470520** | --- |
|  | --- | AY036172 | --- | --- | --- | AF505645 |
| *Monsonia vanderietiae* | South Africa, Fish River; Aldasoro 9088 | --- | **KX470550** | --- | --- | **KX470465** |
|  | --- | --- | --- | EU922459 | --- | --- |
|  | --- | AF167150 | --- | --- | AF167150 | --- |
| *Pelagonium abrotanifolium* | --- | --- | --- | JQ479110 | JQ479167 | --- |
|  | --- | --- | --- | --- | --- | AF256592 |
| *Pelagonium exstipulatum* | --- | --- | --- | JQ479087 | JQ479144 | --- |
|  | South Africa, Little Karoo; Bakker STEU1656 (STEU) | Z95284 | **KY630161** | --- | --- | Z95263 |

**Table S9.** Specific primer pairs and conditions of the polymerase chain reaction for the amplification of DNA markers used in *Monsonia*.

| **Marker** | **Primer Sequence** | **Author primer’s** | **Pre-treatment** | **Treatment** | **Final stage** |
| --- | --- | --- | --- | --- | --- |
| *trn*L*^UAA^*_F | CGA AAT CGG TAG ACG CTA CG | Taberlet *et al*., 1991 | 1min at 96ºC | 30 cycles of 1min at 96°C,  1min at 50°C,  2min at 72°C | 10min at 72°C |
| *trn*F*^GAA^*_R | ATT TGA ACT GGT GAC ACG AG |
| *trn*S^GCU^-F | GCC GCT TTA GTC CAC TCA GC | Hamilton, 1999 | 1min at 96ºC | 30-40 cycles of 45 s at 96ºC,  1min 30s -2min at 45ºC-54ºC,  1min 30s – 2min at 72º C | 10min at 72°C |
| *trn*G^UCC^-R | GAA CGA ATC ACA CTT TTA CCA C |
| *rbc*L^1^-F | ATG TCA CCA CAA ACA GAA ACT AAA GC | Olmstead *et al*., 1992 | 1min at 95ºC | 30 cycles of 45 s- 1min at 95ºC,  30s - 1min at 48-50ºC,  2min at 72º C | 10min at 72°C |
| *rbc*L^724^-R | TCG CAT GTA CCT GCA GTA GC |
| *mat*K ^5^-F | GTA TCG CAC TAT GTA TCA TTT GA | Johnson & Soltis, 1995 | 3min at 95ºC | 35 cycles of 30s at 95°C,  4s at 47°C,  1min 30s at 72°C | 10min at 72°C |
| *mat*K*^C2M^*-R | CAC ACG GCT TTC CCT ATG TAT AC | Haberle *et al*., 2009 |
| *ITS*^1A^-F | TCC GTA GGT GAA CCT GCG G | White *et al*., 1990 | 5min at 95ºC | 38 cycles of 30 s at 95ºC,  30s at 52ºC,  1min at 72º C | 10min at 72°C |
| *ITS*^4^*-*R | TCC TCC GCT TAT TGA TAT GC |

**Supplementary Information**

**Opposite trends in the genus *Monsonia* (Geraniaceae): specialization in the African deserts and range expansions throughout eastern Africa.**

Sara García-Aloy*^1^, Isabel Sanmartín^2^, Gudrun Kadereit^3^, Daniel Vitales^1^, Ana María Millanes^4^, Cristina Roquet^5^, Pablo Vargas^2^, Marisa Alarcón ^1^† and Juan José Aldasoro^1,4^*†


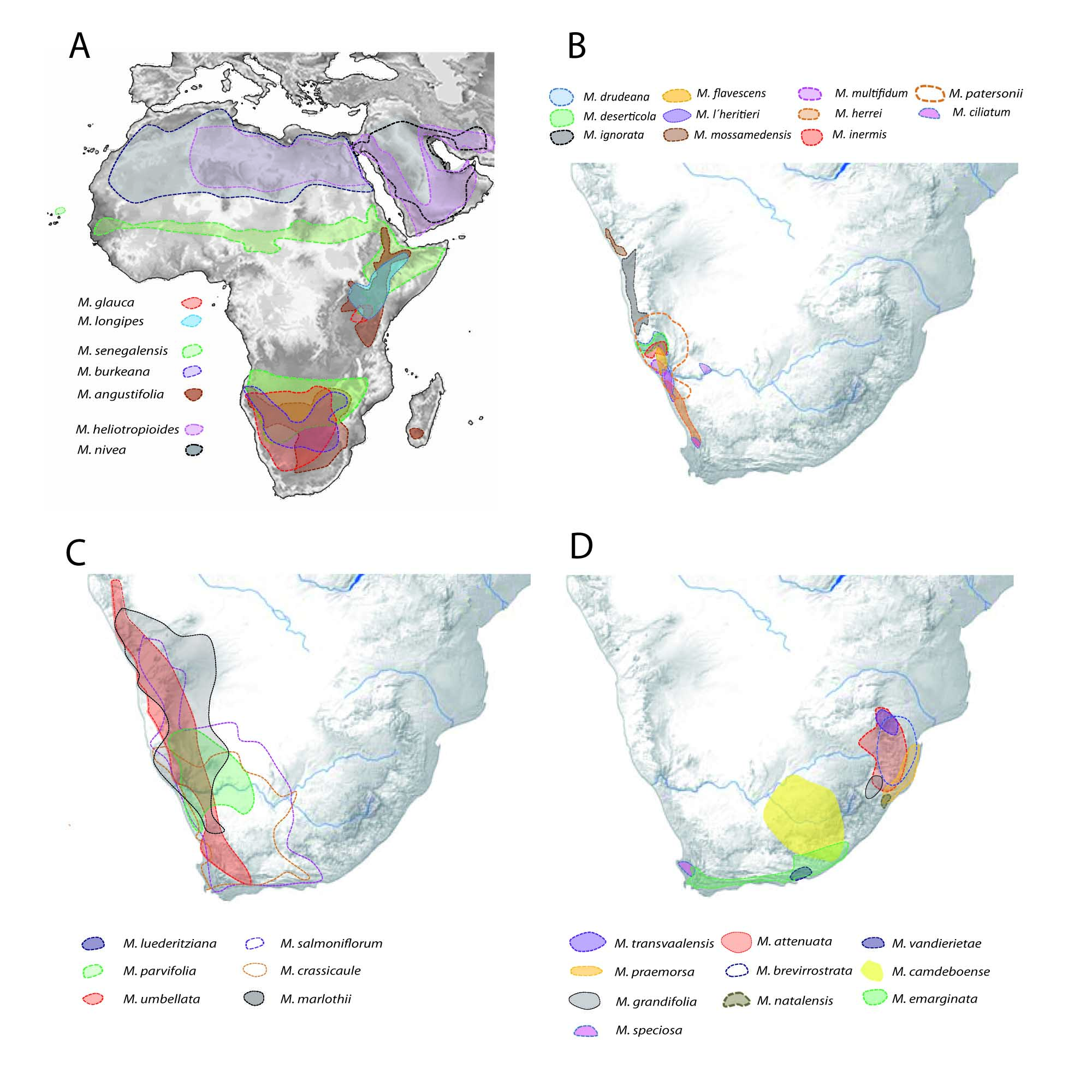


**Figure S1**. Distribution of *Monsonia* species: ( A) species with extensive distributions across the eastern corridors or in northern Africa plus Arabia and western Asia (subclades a, c and f); (B) species restricted to small areas in the Namib Desert, the Succulent Karoo and other close parts of South Western Africa Namib Desert, the Succulent Karoo, and the Nama Karoo (subclades b, d and g); (C) species with large distributions in Southern Africa: (subclades b and g), and (D) species centred in the Natal-Drakensberg or in the Cape (subclades e, f and g). Maps were created using software Adobe Illustrator CS4 (https://www.adobe.com)


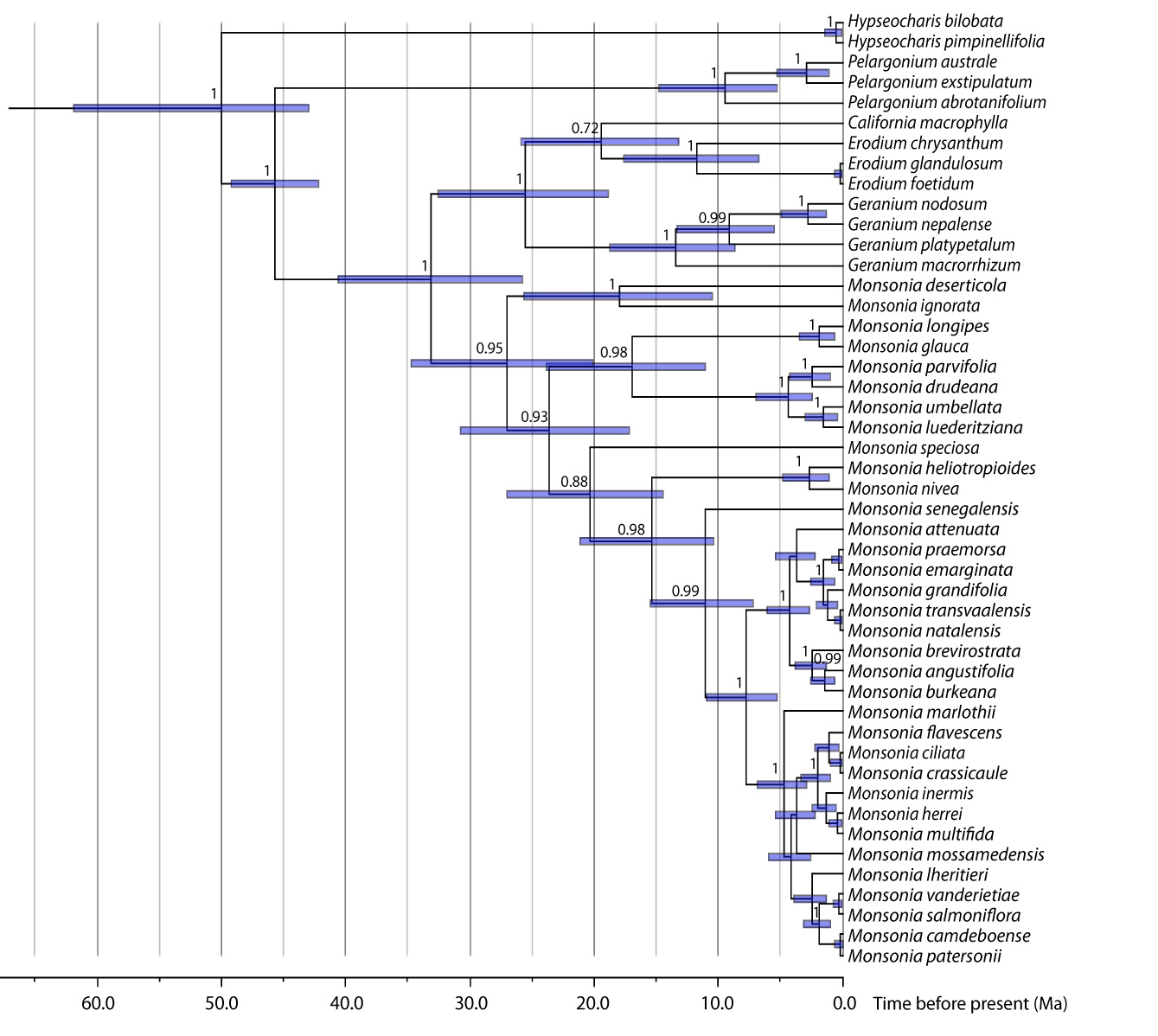


**Figure S2.** Phylogenetic tree of *Monsonia* using the ITS dataset.


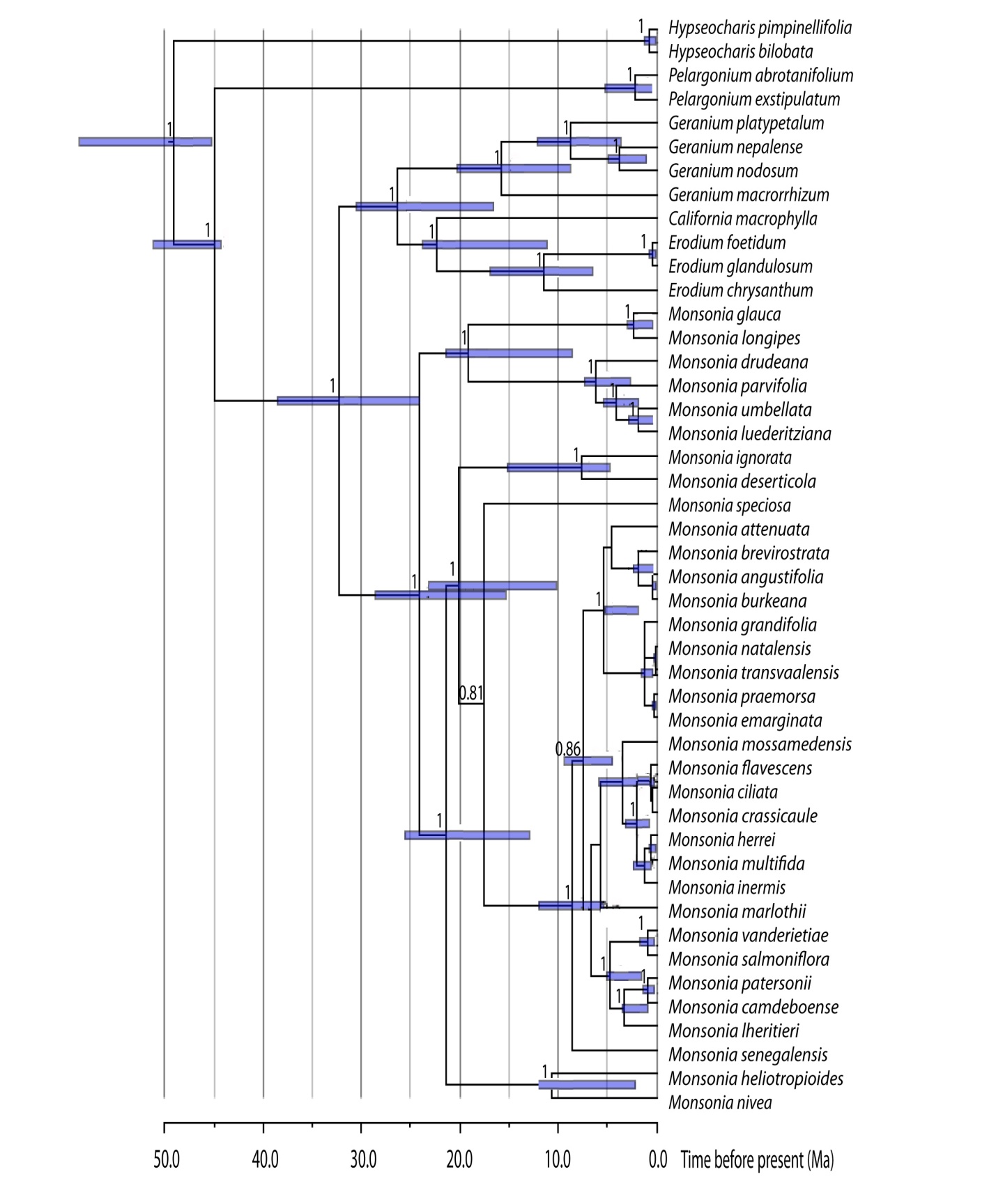


**Figure S3.** Phylogenetic tree of *Monsonia* using the plastid dataset.


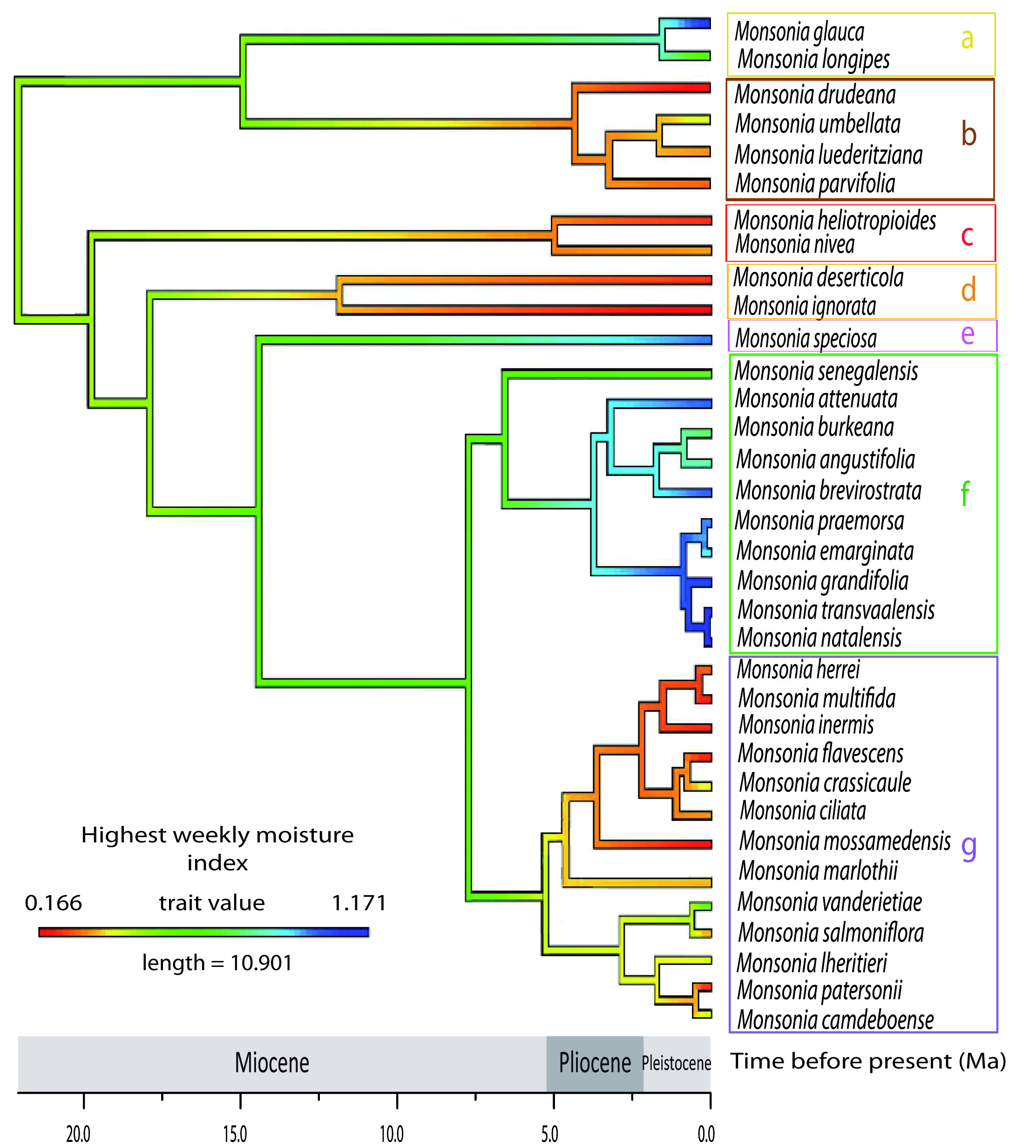


**Figure S4.** MCC tree of *Monsonia* with shifts in highest weekly moisture index inferred using the R package phytools using the Ornstein–Uhlenbeck continuous evolutionary model.


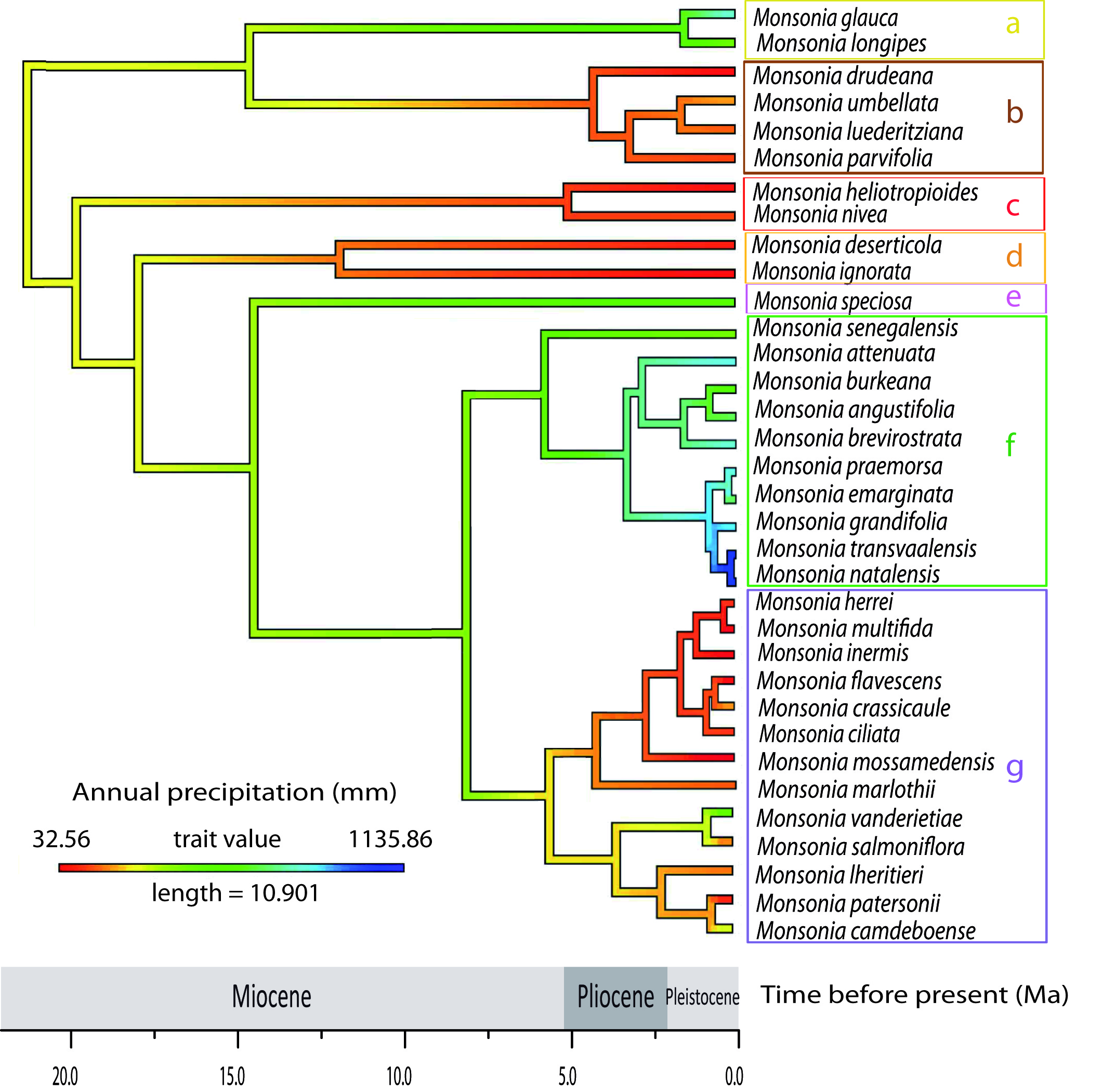


**Figure S5.** MCC tree of *Monsonia* with shifts in annual precipitation inferred using the R package phytools using the Ornstein–Uhlenbeck continuous evolutionary model.


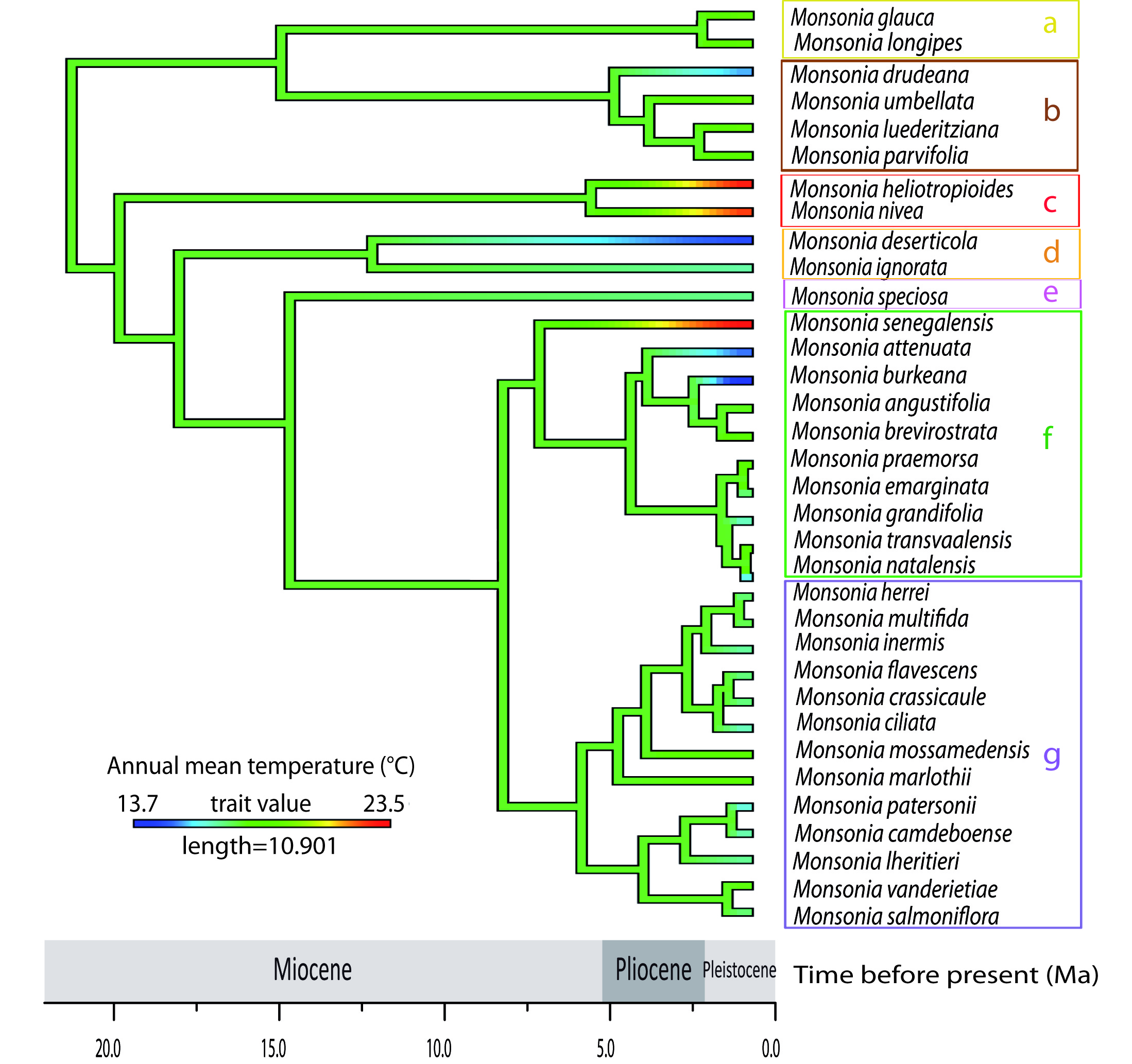


**Figure S6.** MCC tree of *Monsonia* with shifts in annual mean temperature using the R package phytools using the Ornstein–Uhlenbeck continuous evolutionary model.


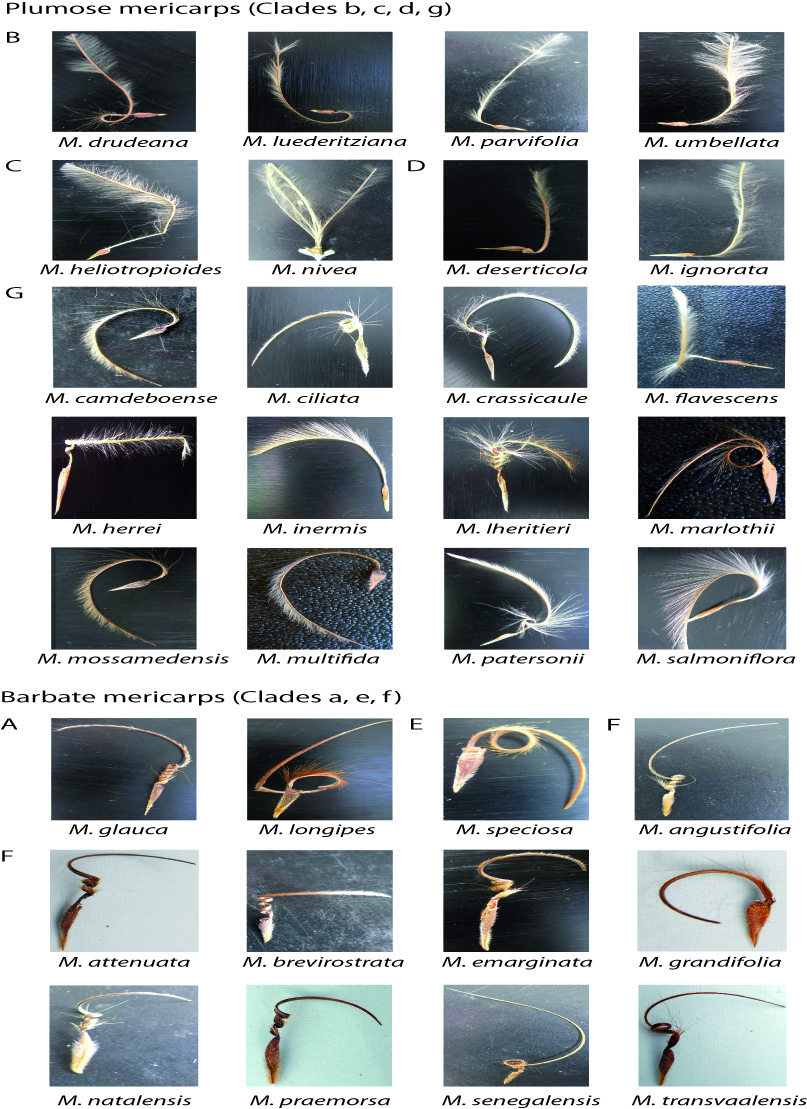


**Figure S7.** Ripe mericarps in *Monsonia* showing differences between plumose and barbate states. All photographs were taken by the co-authors.


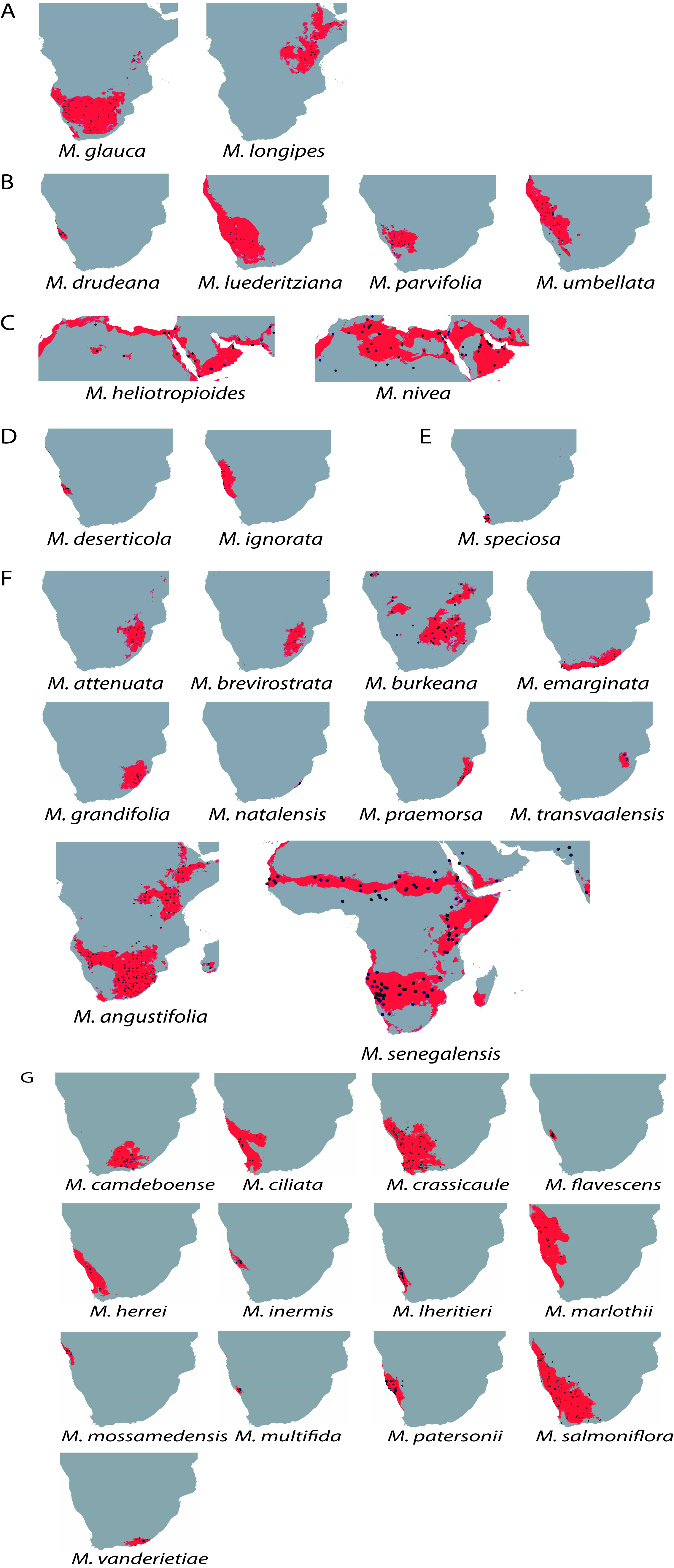


**Figure S8.** Species distribution models (SDMs) of *Monsonia* species as inferred by Maxent. Maps created with the software ArcMap v. 10.1. The average distribution model under current climatic conditions is shown. Occurrence records are shown as black dots in the current maps.
